# Evaluating Novel Direct Injection Liquid Chromatography–Mass Spectrometry Method and Extraction-Based Workflows for Untargeted Lipidomics of Extracellular Vesicles

**DOI:** 10.1021/acs.jproteome.5c00156

**Published:** 2025-08-12

**Authors:** Michał Młynarczyk, Felicja Gajdowska, Jorge Matinha-Cardoso, Paulo Oliveira, Paula Tamagnini, Mariusz Belka, Jagoda Mantej, Danuta Gutowska-Owsiak, Weronika Hewelt-Belka

**Affiliations:** † Department of Analytical Chemistry, Faculty of Chemistry, Gdańsk University of Technology, Gdańsk 80-233, Poland; ‡ Laboratory of Experimental and Translational Allergology and Pneumology, Medical University of Gdańsk, Gdańsk 80-214, Poland; § MCbiology Doctoral Program, ICBAS–School of Medicine and Biomedical Sciences Abel Salazar, University of Porto, Porto 4050-313, Portugal; ∥ CIIMAR–Interdisciplinary Centre of Marine and Environmental Research, University of Porto, Matosinhos 4450-208, Portugal; ⊥ I3S–Instituto de Investigação e Inovação em Saúde, University of Porto, Porto 4200-135, Portugal; # Department of Biology, Faculty of Sciences, University of Porto, Porto 4169-007, Portugal; ¶ Department of Pharmaceutical Chemistry, Medical University of Gdańsk, Gdańsk 80-416, Poland; ∇ Laboratory of Experimental and Translational Immunology, Intercollegiate Faculty of Biotechnology of University of Gdańsk and Medical University of Gdańsk, University of Gdańsk, Gdańsk 80-307, Poland

**Keywords:** lipidomics, extracellular vesicles, lipid extraction, LC-MS, liquid–liquid extraction, solid-phase extraction

## Abstract

Lipids of extracellular vesicles (EVs) are attracting attention due to their crucial biological functions and potential roles in processes such as carcinogenesis. This study compares three commonly used lipid extraction techniques, i.e., liquid–liquid extraction, single-phase extraction, and solid-phase extraction, with a novel direct injection liquid chromatography–mass spectrometry (DI-LC-MS) workflow tailored to EV lipidomics. In the DI-LC-MS approach, EVs are disrupted and released directly in the chromatographic system, enabling the analysis of lipids without a prior extraction step. The applicability of the DI-LC-MS workflow was demonstrated by profiling lipids in mammalian and bacterial EVs. The lipidome coverage and high precision of the DI-LC-MS method (coefficient of variation of peak area lower than 20% for all the identified lipids) enabled identification of differences in lipid profiles of EV samples. The column used in the DI-LC-MS method exhibited a sufficient lifespan and stability for comparative lipidomic studies. Lipidome coverage, lipid species distribution, and precision varied across the studied workflows; our findings highlight the strengths and limitations of these methods. The DI-LC-MS emerges as a sustainable alternative for EV lipidomic studies by eliminating the need for sample preparation and reducing analysis time, solvent use, and chemical noise while requiring less than 1 μL of sample.

## Graphical abstract

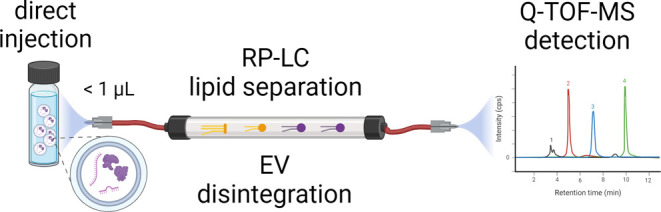

## Introduction

Extracellular vesicles (EVs) are lipid-rich particles produced by eukaryotic and prokaryotic cells, surrounded by a lipid bilayer and carrying bioactive molecules, such as proteins, lipids, and nucleic acids, including miRNA, mRNA, long-noncoding RNA, and even DNA.^1^ Lipids are essential components of EVs, as they form the structural basis of their membranes and likely play additional roles as signaling molecules. However, our comprehension of the EV lipid composition and its biological significance remains incomplete, and this topic has recently gained increased attention.^2–4^

Mass spectrometry enables comprehensive studies of the EV lipid composition.^2,4–11^ However, EV lipidomics presents significant analytical challenges, such as limited sample size (especially for the EVs obtained from primary cells), low lipid concentration, and matrix effects that hinder detection sensitivity. Conventional EV lipidomics workflows rely on lipid extraction to isolate lipids and to remove interfering compounds, such as proteins that can accumulate on the LC column or MS inlet, affecting chromatography performance and reducing instrument sensitivity. Among these, liquid–liquid extraction (LLE) is the most widely used, employing solvent systems like chloroform/methanol or methyl *tert*-butyl ether/methanol/water to disintegrate membranes, precipitate proteins, and isolate lipids.^7,11–13^ Alternatively, single-phase extraction with protein removal by precipitation^14,15^ or solid-phase extraction (SPE)^16^ has been utilized, but their applicability to EV lipidomics remains unexplored. Although effective, extraction-based workflows are time-consuming and require large sample volumes as well as the use of hazardous solvents, such as chloroform. Moreover, the extraction process itself can influence the lipidome coverage, lipid recovery, and repeatability. Comparative studies on how the extraction method impacts EV lipidome coverage remain scarce despite their critical role in lipidomics data quality.

To address the limitations of conventional workflows, we demonstrate the applicability of direct injection liquid chromatography coupled with a mass spectrometry (DI-LC-MS) method tailored for untargeted EV lipidomics. Direct dissolution of small-volume samples is a recognized practice in LC–MS workflows, particularly for small molecules in relatively simple matrices. This approach reduces the required sample volume and minimizes contamination risks associated with extraction chemicals and materials.^17–19^ While direct injection LC–MS has been widely used for simpler matrices, its application to complex biological systems such as cells represents a significant advancement.^20,21^ To our knowledge, this study represents one of the first applications of the direct injection LC–MS approach in EV lipidomics, offering a fast, sustainable, and efficient alternative to extraction-based workflows.

This study compares three commonly used lipid extraction methods: LLE, single-phase extraction, and SPE with a novel DI-LC-MS workflow, focusing on their respective advantages and limitations in EV lipidomics. By evaluating lipidome coverage, precision, and the percentage distribution of the lipid species and by comparing EV lipid profiles across different sources, we aim to provide researchers with a practical framework for selecting the most suitable approach for EV lipidomics.

## Materials and Methods

### Reagents for LC–MS Analysis and Lipid Extraction

Methanol and 2-propanol hypergrade for LC–MS, chloroform for liquid chromatography, ammonia solution 25% for HPLC, formic acid ≥98% grade, ammonium formate LiChropur ≥99%, and phosphate buffer saline (PBS) were purchased from Sigma-Aldrich (St. Louis, MO, USA). Deionized water was purified by an HLP5 system (Hydrolab, Wiślina, Poland) and used for the preparation of aqueous solutions. A lipid standard solution EquiSPLASH LIPIDOMIX was purchased from Avanti Polar Lipids (Birmingham, AL, USA).

### Samples

Fetal bovine serum EVs (FBSEV) and EVs derived from cultured cyanobacteria cells (CEV) were isolated by ultracentrifugation (for further details, see below). EVs from human plasma (lyophilized exosomes from Plasma of Healthy donors, no. M1040-100-2) were purchased from AMSBIO (Abingdon, UK). According to AMSBIO’s policies, all samples were collected and provided in compliance with appropriate ethical standards. Cyanobacterial EVs and human plasma EVs were resuspended in deionized water before DI-LC-MS analysis.

### Isolation of Fetal Bovine Serum EVs

Fetal bovine serum (Sigma-Aldrich (St. Louis, MO, USA)) was mixed 1:1 with the RPMI-1640 or DMEM medium (Sigma-Aldrich (St. Louis, MO, USA)) and ultracentrifuged for 19 h at 100 000 × *g* at 4 °C (Optima L-90K ultracentrifuge, Beckman; 50.2 Ti ultrarotor, Beckman). Afterward, the pellet was resuspended in cold phosphate buffer solution (PBS) (filtered by a 0.1 μm size filter). Isolated vesicles were kept at −80 °C until further experiments. FBSEVs were characterized according to the Minimal information for studies of extracellular vesicles (MISEV2023).^22^ The details of nanoparticle tracking analysis (NTA), transmission electron microscopy (TEM), and Western blotting methods are shown in the Supporting Information file.

### Isolation of Cyanobacterial EVs

For EV isolation, a previously described protocol using an ultracentrifugation method was followed.^23,24^ In brief, a cell-free extracellular medium was initially concentrated by centrifugal ultrafiltration using membranes with a molecular weight cutoff of 100 kDa (Pall). Then, the concentrated medium was ultracentrifuged for 3 h at 100000 × *g* at 4 °C, and the supernatant was discarded, while the sediment was suspended in sterile-filtered PBS solution. The solution containing cyanobacterial EVs was then stored at −80 °C until characterization and lyophilization. To assess EV morphology, size, and amount and further characterize isolated vesicles, samples were analyzed by transmission electron microscopy (TEM) and nanoparticle tracking analysis (NTA) (NanoSight) and by inspecting the protein and lipopolysaccharide (LPS) profiles on SDS-polyacrylamide gels, as described elsewhere.^23^

### Lipid Extraction Procedures

EV lipids were extracted using previously described methods, with slight modifications. Details of applied methods (liquid–liquid extraction,^25^ single-phase extraction,^26^ and solid-phase extraction^27^) are provided below. An extraction blank was prepared for each set of samples using water instead of the sample. Samples were extracted in triplicate, and each extract was analyzed in LC–MS triplicate.

### Liquid–Liquid Extraction

5, 10, or 20 μL of FBSEV sample was transferred into a borosilicate-glass tube with a PTFE cap, followed by the addition of water to the volume of 225 μL. Next, 950 μL of a chloroform/methanol mixture (1/2; *v/v*) was added. Samples were vortexed vigorously for 10 s. Then, 310 μL of water and 310 μL of chloroform were added to each tube. Samples were vortexed vigorously for 30 s and centrifuged at 3234 × *g* at 4 °C to separate the aqueous and organic phases. The lower phases were transferred to a new borosilicate glass tube by a glass Pasteur pipet. 490 μL of supernatants was transferred to new glass tubes and evaporated under a nitrogen stream at 30 °C. The dried extracts were stored at −80 °C overnight. Before LC–MS analysis, the dry extracts were dissolved in 50 μL of methanol and transferred into chromatographic vials with inserts.

### Single-Phase Extraction

1, 5, or 10 μL of FBSEV sample was transferred into Eppendorf tubes, followed by the addition of 49, 95, and 90 μL of methanol containing 1% formic acid. The samples were then vortexed vigorously for 30 s and centrifuged at 15557 *x g* at 4 °C. The supernatant was collected using a glass Pasteur pipet and transferred into chromatographic vials with inserts.

### Solid-Phase Extraction

20 or 50 μL of FBSEV sample was transferred into Eppendorf tubes, followed by the addition of water to the volume of 100 μL. Then, 900 μL of methanol containing 1% formic acid was added to each tube, vortexed vigorously for 30 s, and centrifuged at 12857 × *g* at 4 °C. The supernatants were collected and transferred to new Eppendorf tubes. Afterward, 900 μL of each supernatant was loaded onto a HybridSPE-Phospholipid cartridge (bed weight, 30 mg) (Supelco, Sigma-Aldrich, St. Louis, MO, USA) and washed in sequence with 2 mL of methanol, 2 mL of 2-propanol, and 2 mL of methanol. Finally, the lipids were eluted with 2 mL of 5% ammonia in methanol. The extracts were evaporated under a nitrogen stream at 30 °C. The dried extracts were stored at −80 °C overnight. Before LC–MS analysis, the dry extracts were dissolved in 50 μL of methanol and transferred into chromatographic vials with inserts.

### Lipid Profiling by RP-LC-MS

Lipid profiling was carried out with the use of an LC-Q-TOF-MS system: an Agilent 1290 LC system equipped with a binary pump, an online degasser, an autosampler, and a thermostated column compartment coupled to a 6540 Q-TOF-MS with a Jet Stream Technology Ion Source (Agilent Technologies, Santa Clara, CA, USA). The Kinetex EVO C18 2.1 × 100 mm, 1.7 μm (Phenomenex, Torrance, CA, USA) with a 0.2 μm in-line filter was used for the separation of lipids. The column temperature was set to 60 °C. The mobile phase used for DI-LC-MS (chromatographic conditions 1) analysis consisted of component A: 5 mM ammonium formate in water/methanol (2/8; *v/v*) and component B: 5 mM ammonium formate in 2-propanol. The flow rate of the mobile phase was set to 0.3 mL/min. The gradient elution program started with 20–40% B from 0 to 20 min, 40–60% B from 20 to 40 min, 60–100% from 40 to 45 min, and 100% B from 45 to 55 min, followed by 10 min equilibration. The mobile phase used for the analysis of lipid extracts (chromatographic conditions 2) consisted of component A: 5 mM ammonium formate in water/methanol (2/8; *v/v*) and component B: 5 mM ammonium formate in water/methanol (1/99; *v/v*). The flow rate was set at 0.5 mL/min. The gradient elution program started with 20–100% B from 0 to 15 min and 100% B from 15 to 30 min, followed by 10 min equilibration. Electrospray ionization (ESI) source was operated in positive ionization mode. Data were collected in SCAN mode in a range from 200 to 1700 *m*/*z* in a high-resolution mode (4 GHz). The fragmentor and capillary voltage were set to 120 and 5000 V, respectively. Other parameters were set as follows: nebulizer gas pressure of 35 psi, drying gas flow rate of 10 L/min, and temperature of 300 °C. The Q-TOF detector was calibrated daily prior to sample analysis. The MS/MS analyses were performed using identical chromatographic and ion source conditions. The collision energy was set to 35 and 80 V. The two most abundant ions were selected for fragmentation and excluded for the next 0.3 min. The MS/MS spectra were acquired in the *m*/*z* range of 50–1700. The lipid extracts were kept in the autosampler at 10 °C during the batch run.

### Data Treatment

The Molecular Feature (MF) data set for the untargeted approach was generated from the raw LC–MS data in the Agilent MassHunter Workstation Profinder 10.0 software (Agilent Technologies, Santa Clara, CA, USA) using the following parameters: ion threshold, >1000 counts; ion type, H^+^, isotope model, common organic (no halogens); charge state range, 1–2; MFE score, >75. The obtained data set was further treated in Mass Profiler Professional 15.1 software (Agilent Technologies, Santa Clara, CA, USA). The MFs present in extraction blank were removed from the data set and filtered based on frequency (MF present in at least 4 of 5 samples in a group for evaluation of DI-LC-MS and 3 of 3 samples in the group for the extraction methods). MFs were aligned with the following parameters: alignment slope = 0.0%; alignment intercept = 0.2 min; mass tolerance slope = 20.0 ppm; intercept = 2.0 mDa. Missing values were exported as missing. Data was exported to.csv files. The CV of peak volume (for untargeted data with MFs) and the CV of peak area (for identified lipids) were calculated using Microsoft Excel 2016 software (Microsoft Corporation, Redmond, WA, USA). For the assessment of the number of Molecular Features, we included all the peaks integrated as [M + H]^+^ without differentiating adducts and fragment ions, which were detected as separate molecular features (MFs).

The data set for the comparative analysis of relative amounts of identified lipids was generated with the use of Agilent MassHunter Workstation Profinder 10.0 software (Agilent Technologies, Santa Clara, CA, USA) using the following parameters: ion threshold, >1000 counts; ion type, H^+^ for PC, PC-O, SM and NH_4_^+^, Na^+^, neutral loss of water for TG, DG, and CE; isotope model, common organic (no halogens); charge state range, 1–2; MFE score, >75. Next, the relative amounts of identified lipids were calculated in Microsoft Excel by dividing the peak area of lipid species by the sum of lipid species belonging to the same class. The statistical analysis was performed in Metaboanalyst 6.0 (https://www.metaboanalyst.ca/). For the volcano plot, *p*-value threshold and fold change cutoff were set to 0.05 and 2, respectively.

The peak areas, peak width, and peak symmetry for column stability were assessed in the Agilent MassHunter Qualitative 10.0 software (Agilent Technologies, Santa Clara, CA, USA) by automated peak integration. The symmetry of the peak was computed as the ratio between the front half-width and the back half-width.

### Lipid Identification

Lipid identification was carried out by a search of the Human Metabolome Database based on an accurately measured *m*/*z* value (10 Δppm tolerance) and interpretation of the MS/MS spectra. Identification resulted in the determination of the lipid class, number of carbon atoms, and number of unsaturated bonds in fatty acid residues as well as the presence of ether bonds instead of ester bonds in the lipid structure. Lipid species with ether-linked substituents were not differentiated regarding ether and vinyl ether bonds in position *sn*-1. The diagnostic ions for the lipid class confirmation were as follows: *m*/*z* 184.0726 for confirmation of the SM and PC identity, *m*/*z* 369.3536 for confirmation of CE. The cyanobacterial EV lipids were identified accordingly to the previously published procedure.^28^

## Results

### Lipidome Coverage of the DI-LC-MS Method

To demonstrate the feasibility of profiling the EV lipidome through a DI-LC-MS approach, we utilized reversed-phase (RP) chromatography with a C18-modified stationary phase. The high organic solvent content in the chromatographic system (80% methanol/water (4/1; *v/v*) and 20% 2-propanol) was applied to disrupt EV structure, facilitating the separation and detection of lipid species without prior extraction. We adapted the previously developed chromatographic conditions enabling the separation and ionization of a wide range of EV lipids.^25^ EV samples obtained through ultracentrifugation from cyanobacterial cells (CEV), human plasma (HPEV), and fetal bovine serum (FBSEV) were injected into the chromatographic column without a lipid extraction step (see Figures S1and S2 for detailed characterization of the isolated cyanobacterial and fetal bovine serum EV samples, respectively).

Using this setup, we successfully profiled EV lipidomes from three distinct sources, demonstrating the DI-LC-MS method’s ability to disintegrate EV membranes directly in the chromatographic system as shown in the representative chromatograms in Figure 1A–C. Representative chromatograms in positive ionization mode showed lipid profiles for FBSEVs (injection volume 0.1 μL, 1.25 × 10^8^ particles; Figure 1A), HPEVs (injection volume 0.5 μL, 1 × 10^9^ particles; Figure 1B), and CEVs (injection volume 1 μL, 1 × 10^9^ particles; Figure 1C). The method provided separation and detection of lipid species across all of the EV types, spanning both polar and nonpolar lipid classes.

**Figure 1:**
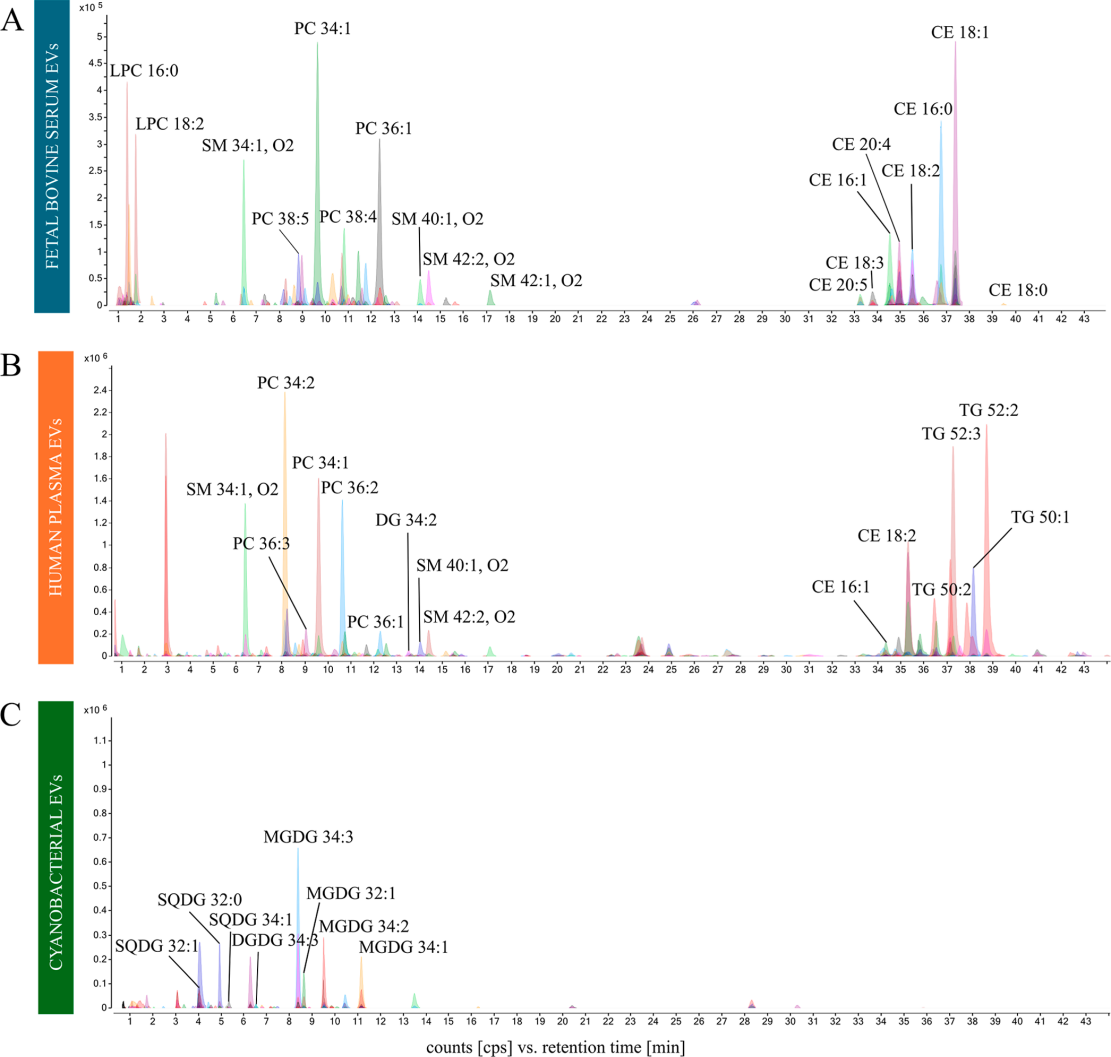
Representative extracted compound chromatograms (ECCs) obtained by DI-RP-LC-Q-TOF-MS of EVs of fetal bovine serum (FBSEV) (A), human plasma (HPEV) (B), and cyanobacteria (CEV) (C). Chromatographic conditions 1 were utilized for this study.

For HPEVs, 483 molecular features (MFs) were detected, with glycerophospholipids (GP), sphingolipids (SP), glycerolipids (GL), and cholesteryl esters (CE) identified among them. Similarly, 174 MFs were detected in FBSEVs, spanning lipid classes comparable to those of HPEVs. In contrast, CEVs displayed a distinct lipid profile with 171 MFs dominated by cyanobacteria-specific membrane lipids such as monogalactosyldiacylglycerol (MGDG), digalactosyldiacylglycerol (DGDG), and sulfoquinovosyldiacylglycerol (SQDG). Characteristics of the identified lipids along with their percentage distribution in samples of CEV, HPEV, and FBSEV are presented in Tables S1–S3 (Supporting Information).

Although HPEVs exhibited a greater number of total MFs compared to FBSEVs, the diversity in the identified lipid species was modest; 79 species were identified in HPEVs and 70 in FBSEVs, with 66 species shared between both EV types. The qualitative differences included mainly triacylglycerols (TGs) and diacylglycerols (DGs) species detected in HPEV samples and unique lysoglicerophosphocholines (LPCs) in FBSEV samples. Further analysis of lipid species distribution within major classes revealed distinct profiles for HPEVs and FBSEVs. A comparative analysis of the lipid profiles for HPEV and FBSEV identified statistically significant differences (*p* < 0.05, FC > 2) in the relative abundance of 33 out of 66 lipid species, as shown in Figure 2A. The complete list of significantly different lipids is provided in Table S4 (Supporting Information). Specifically, PC and its ether analogs, as well as CE species, varied significantly between the two EV types (Figure 2C–E); e.g., the most abundant lipid species was PC 34:1 (20.8% on average) for the PC class in FBSEVs, while PC 34:2 dominated in HPEVs (39.6%). Interestingly, PC 34:2 represented only 1.35% of all the PCs in FBSEVs, indicating a marked difference in the lipid composition. Additionally, PC species containing long-chain polyunsaturated fatty acids (LC-PUFAs), such as PC 40:5, PC 40:6, and PC 38:5, were more abundant in FBSEVs than in HPEVs. Similarly, LPC species were also enriched in FBSEVs, with LPC 16:0 contributing 7.8% of all the PCs in FBSEVs, compared to only 0.5% in HPEVs.

**Figure 2:**
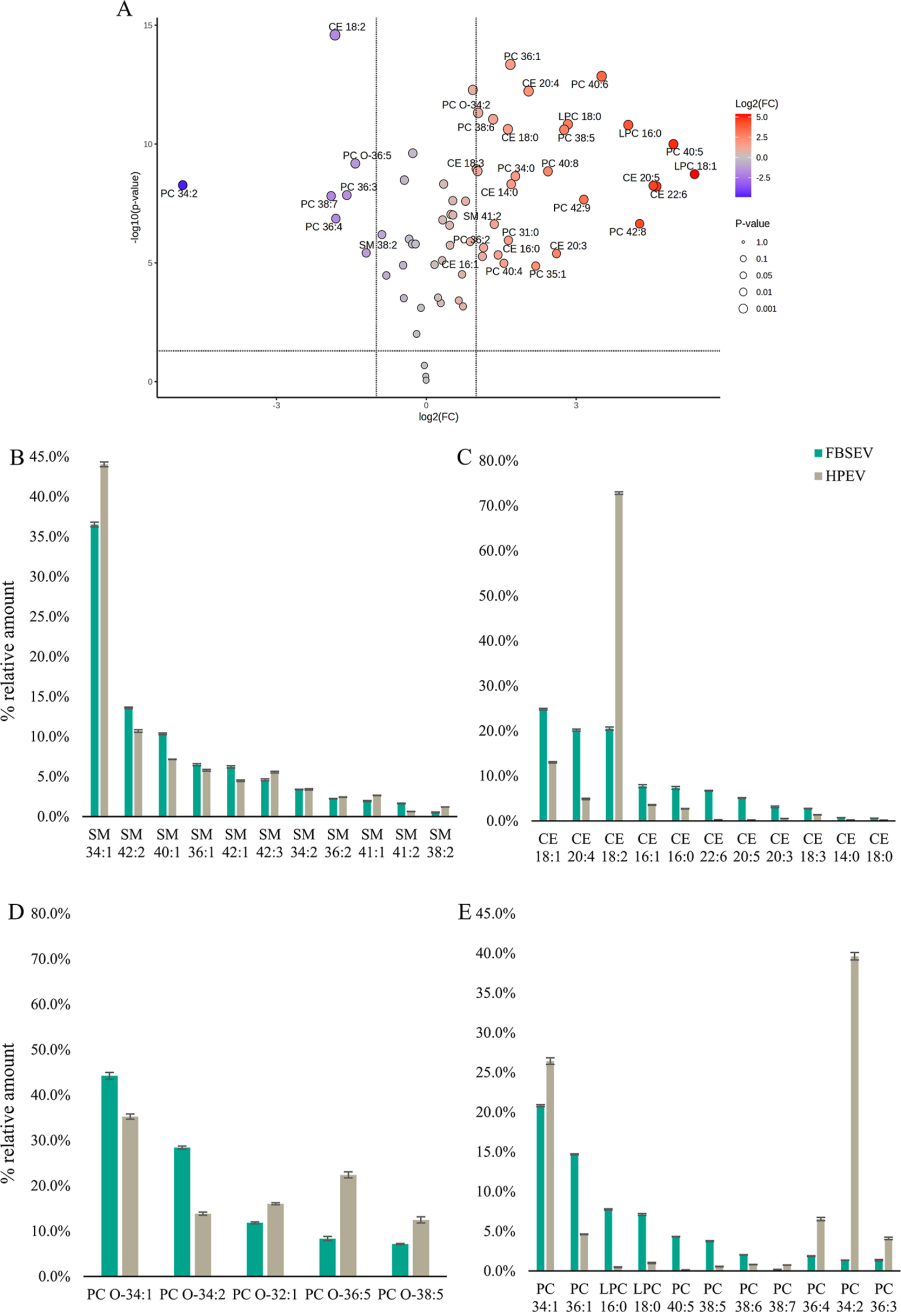
Volcano plot comparing the lipid profiles of human plasma EV (HPEV) and fetal bovine serum EV (FBSEV). Direct comparison of FBSEV/HPEV, *p*-value threshold = 0.05, fold change = 2.0, unpaired analysis. The blue color indicates higher content of lipid species in FBSEVs, while red color indicates those more abundant in HPEVs (A). Average relative amount of the selected PC and its ether analogs, SM and CE species in FBSEV (blue bars) and HPEV (red bars) samples, and error bars correspond to standard deviation (*n* = 5) (B). Precision, MS signal stability, and chromatographic performance of the DI-LC-MS method.

The DI-LC-MS method demonstrated good precision for the lipidomic analysis across the three EV sample types. More than 60% of molecular features in all tested samples exhibited a percent coefficient of variation (%CV) of peak volume below 20%. The total MS signal variation across five replicates (*n* = 5) for each sample type was less than 8% (Supporting Information, Table S5). The % CV of peak area of all identified lipids for CEV, FBSEV, and HPEV was below <20%. These metrics underscore the reliability of the DI-LC-MS method for untargeted EV lipidomics.

Comparison of the total ion chromatograms (TICs) obtained through DI-LC-MS with those from liquid–liquid extraction (LLE) workflows showed a reduced number of contaminant peaks in comparison to LLE extracts (Figure 3). These additional LLE peaks, also observed in the extraction blanks, are likely introduced by chemicals used during the extraction and evaporation steps. Hence, TIC from DI-LC-MS showed significantly reduced chemical noise while maintaining comparable MS signal intensity. Notably, the DI approach required 40 times less sample volume (0.5 μL vs 20 μL for LLE) to achieve similar results, demonstrating its efficiency and suitability for low-volume EV samples.

**Figure 3:**
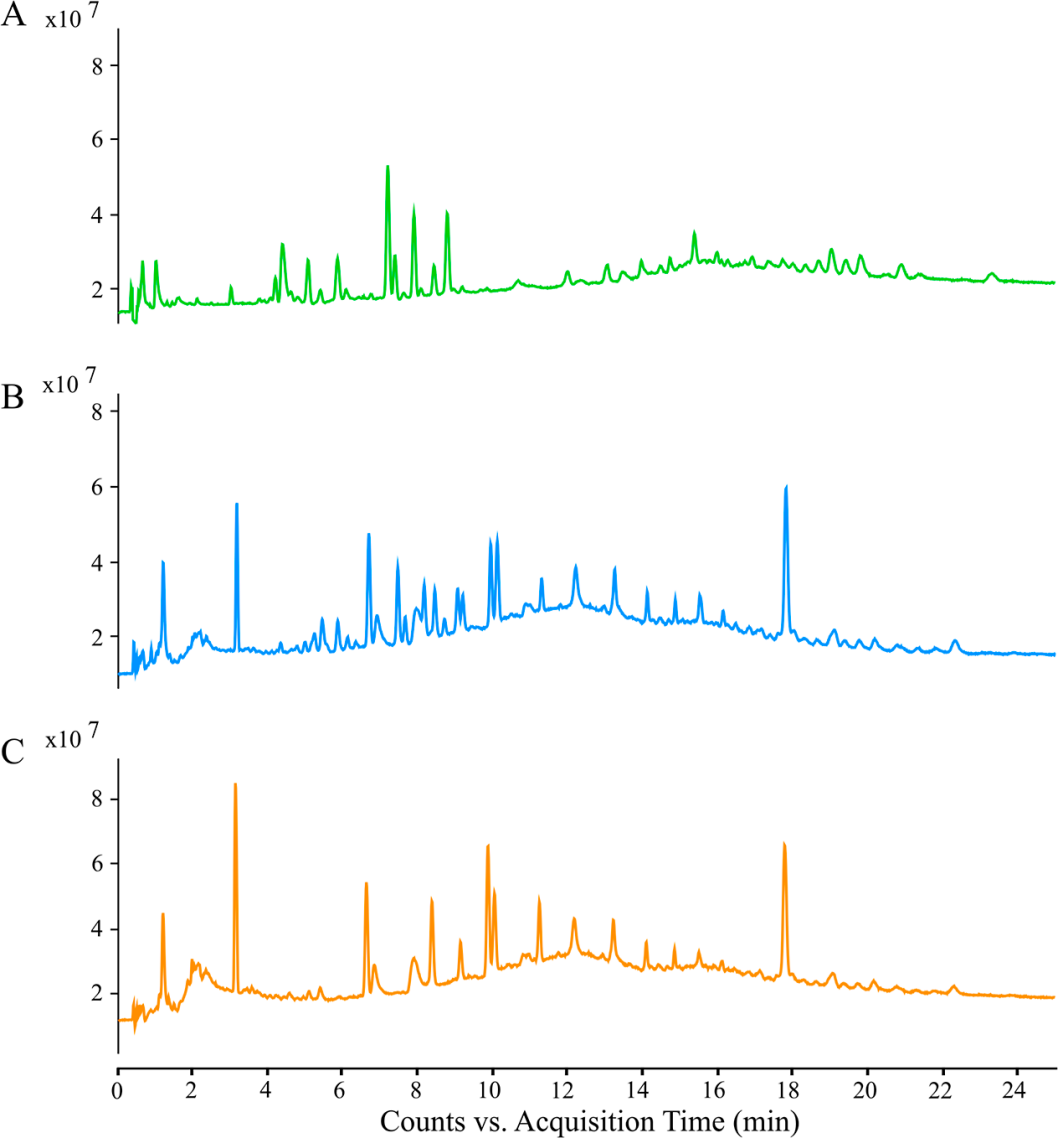
Representative total ion chromatograms (TIC) of the CEV lipids analyzed by DI-LC-MS (A), LC–MS analysis of lipid extract (B), and extraction blank (C) obtained by LLE. Chromatographic conditions 2 were utilized for this study.

In addition to the assessment of lipid profiles, the long-term performance of the chromatographic column was also evaluated, given the complexity of EV samples, which also include polar metabolites, proteins, peptides, and possibly undisintegrated EVs (approximate size 100–200 nm). Such components can cause column clogging, loss of resolution, and a shortened column lifespan. To assess these issues, we performed 30 consecutive injections of intact CEV samples spiked with lipid standards (EquiSPLASH LIPIDOMIX). Peak area and retention time variance, peak width, and symmetry were monitored across the batch (Figure 4A–E). Additionally, as a reference sample, we also injected the lipid standard (EquiSPLASH LIPIDOMIX) at the start, toward the end, and between every of the nine CEV samples.

**Figure 4:**
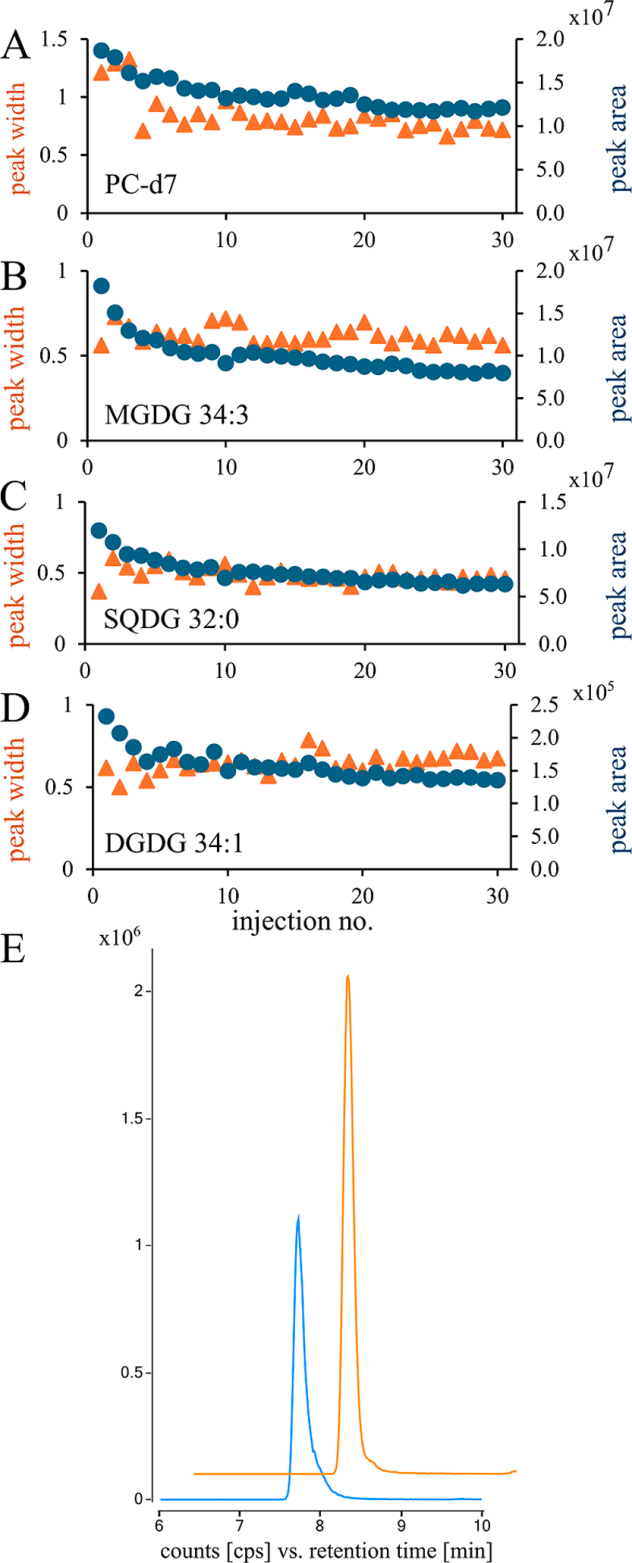
Peak width (orange triangle) and peak area (blue dot) of 15:0–18:1­(d7) PC standard (A), MGDG 34:3 (B), SQDG 32:0 (C), DGDG 34:1 (D) assessed during the sequence of 30 injections of CEV samples spiked with lipid standards. Peaks of MGDG 34:3 acquired in the first (orange) and 30th (blue) CEV run (E).

We observed a more than 2-fold decrease in the peak area for lipid standards in the reference samples after the initial injections of native EV samples; however, signal intensity stabilized after 5–7 injections, depending on the compound (Supporting Information, Table S6). The %CV of peak areas for lipid standards in the spiked EV samples remained below 20%, and the variance further decreased when the initial injections were excluded. A similar trend was observed for lipids originating from the EV sample (Figure 4B–D); however, their peak area slightly decreased throughout the batch. This signal decline could be attributed to vesicle sedimentation at the bottom of the vial over time or partial lipid degradation, as the sequence lasted more than 30 h. Importantly, despite the decrease in the peak area for some compounds, the number of detected lipid species and overall lipidome coverage remained stable across the batch.

The peak width showed acceptable stability, with %CV values below 20% across 30 injections. However, peak symmetry deteriorated progressively over the sequence, as shown in Figure 4E. Despite this loss of symmetry, no significant impact on the precision (expressed as the %CV of the peak area) was observed. The retention time was stable during the batch, with %CV below 1% for the majority of the tested compounds (Table S6). We also did not observe a significant increase in the column backpressure (Supporting Information, Figure S3). These findings highlight that while the column performance declines with prolonged use, the method retains sufficient precision for lipidomic analysis.

### Comparison of the Extraction Methods for EV Lipidome Characterization

Next, we evaluated and compared three widely used extraction methods: liquid–liquid extraction, single-phase extraction, and solid-phase extraction (HybridSPE-phospholipids), focusing on their impact on the lipidome coverage and precision. Specifically, we assessed the total number of detected molecular features, the relative distribution of lipid species within each class, and the precision of each method, as measured by the coefficient of variation of the peak areas for both identified and unidentified lipids. Finally, we compared the performance of these extraction methods to the newly developed DI-LC-MS workflow.

First, we evaluated the impact of the sample volume and FBSEV particle number used for extraction on the number of detected molecular features. The initial concentration of the FBSEV particles was 6.1 × 10^11^ particles/mL, as determined by Nanoparticle Tracking Analysis (2NTA; 2.0 × 10^8^ ± 2.2 × 10^7^ particles/mL after 3000× dilution). Among the tested methods, single-phase extraction using methanol with 1% formic acid showed the greatest sensitivity to sample volume, with a significant reduction in the number of detected MFs at lower volumes (Figure S4). This method also yielded fewer detected lipids overall compared to LLE. In contrast, the number of detected MFs was not significantly influenced by the sample volume for LLE or SPE (Supporting Information, Figure S4)

The LLE enabled isolation of the greatest number of MFs, including both polar phospholipids and nonpolar CEs, as shown in Table 1. Using SPE with a stationary phase containing silica modified with zirconia disabled the isolation of these nonpolar lipid classes. However, many polar compounds were coisolated, which eluted in the earlier region of the chromatogram, especially in the first 5 min of the LC–MS run. Single-phase extraction technique using methanol supplemented with 1% formic acid allowed us to obtain lipidome coverage similar to LLE, but with lower precision. DI-LC-MS enabled the detection of a slightly lower amount of MFs in comparison to the extraction methods; however, it has to be noted that a significantly lower amount of the biological sample was used for the analysis.

**Table 1:** Comparison of the Number of Identified Lipids in the FBSEV Sample Analyzed with Different Methods

	number of particles for analysis	EV sample volume [μL]	number of MFs	number of identified lipids	number identified lipids with %CV < 20%	number of CE	number of PC	number of PC-O	number of SM
DI-LC-MS	1.25 × 10^8^	0.1	174	70	70	12	34	5	19
LLE	2.50 × 10^10^	20	277	75	75	11	42	8	14
SPE	6.25 × 10^10^	50	230	66	66	0	42	8	14
single-phase	1.25 × 10^10^	10	197	75	50	11	42	8	14

The number of identified lipids was comparable between the extraction methods and the DI-LC-MS approach (Table 1). A slightly lower number of PCs and LPCs and their ether analogs (13 LPC and 3 LPC-O detected in lipid extracts vs 8 LPC and 0 LPC-O detected by DI-LC-MS) and a higher number of the SM species (19 SM detected by DI-LC-MS and 14 SM detected in lipid extracts) were detected by the DI-LC-MS method in comparison to extraction-based workflows.

Next, we investigated differences in the relative distribution of lipids obtained with these methods. The percentage distribution of lipids within various lipid classes is presented in the Supporting Information (Tables S7–S9).

Investigation of the results showed a comparable pattern between all tested extraction methods and DI-LC-MS for CE and SM (Figure 5). A different pattern was detected for the PCs obtained with SPE, distinctly from other methods. A higher percent contribution of LPC could be observed for SPE; a slightly different pattern was also observed between DI-LC-MS and extraction methods for CEs, with a lower relative amount of CE 20:4 in comparison to LLE and single-phase extraction.

**Figure 5:**
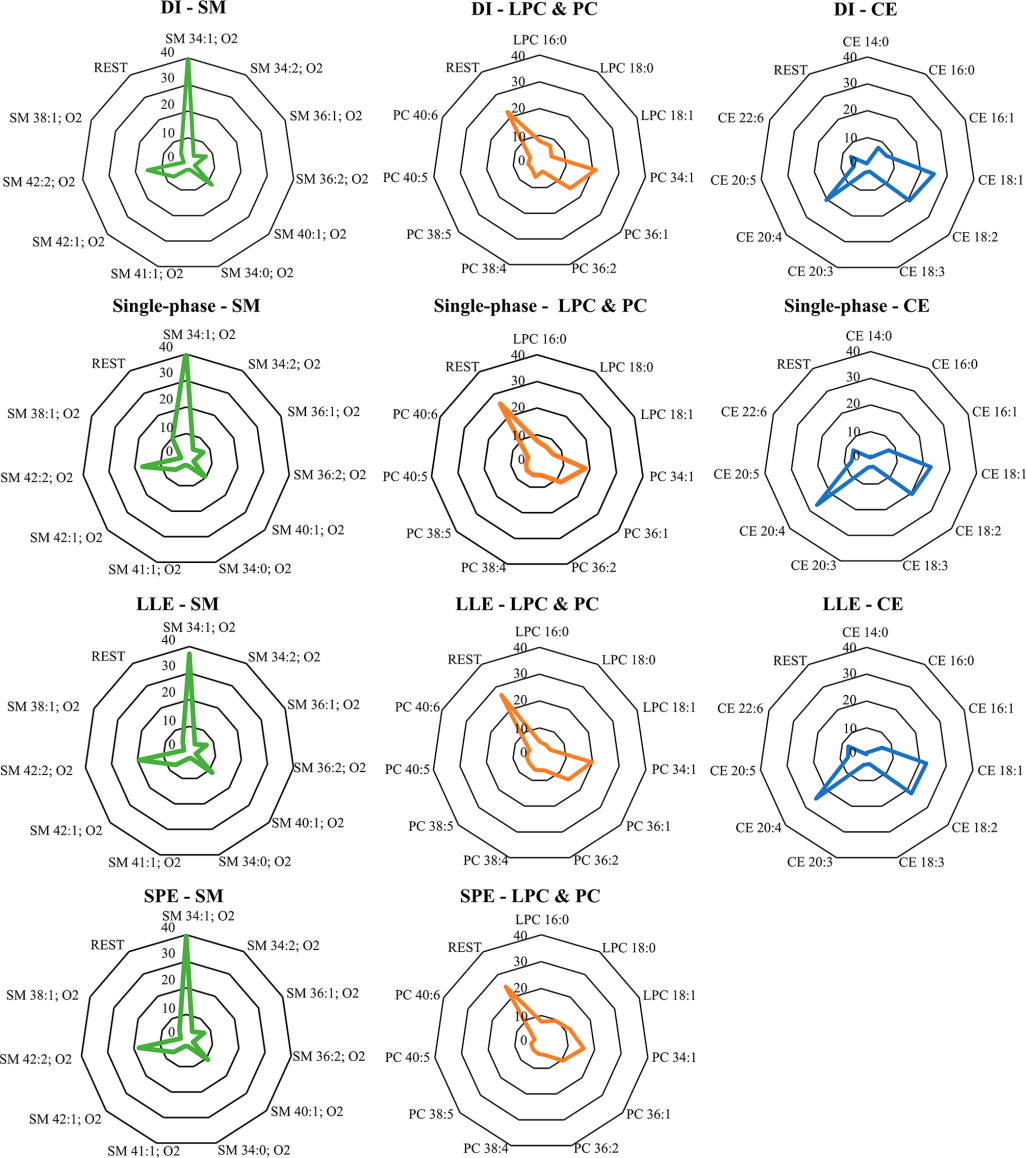
Radar plots showing the percent relative amount of the lipid species within a class of PC, SM, and CE in EVs. Lipid profiles obtained by the DI-LC-MS method, LLE, SPE, and single-phase extraction as labeled. The radar plots were created based on the ten most abundant lipids for DI-LC-MS methods. Additionally, only lipids detected in all of the methods were included for the calculation of percentage distribution to minimize the impact of qualitative differences between the tested samples (Supporting Information, Table S10).

The precision of the applied methods was estimated based on the coefficient of variation of peak areas. The analysis showed that 58%, 60%, and 28% of the detected MFs in the untargeted approach had a CV lower than 20% for LLE, SPE, and single-phase extraction by methanol with 1% formic acid, respectively. Similarly, the highest variance in the total MS signal was observed for the single-phase extraction. When comparing the peak areas of the identified lipids, only the single-phase extraction method was characterized by low precision with only 50 lipids (out of 75) with a peak area %CV lower than 20% (Table 1).

### Comparison of the Proposed Lipidomic Methods Using Green Chemistry Metrics

One of the primary motivations for developing the DI-LC-MS lipidomic method was its alignment with the principles of sustainability and “green chemistry”. Multiple numerical protocols exist to evaluate the environmental impacts of analytical procedures. We employed the blue applicability grade index (BAGI) for this purpose, as it both incorporates classical green principles and considers practical aspects and emphasizes sample preparation.^29^ The BAGI software (https://bagi-index.anvil.app/) effectively differentiated the environmental impact of the four applied procedures (Figure 6).

**Figure 6:**
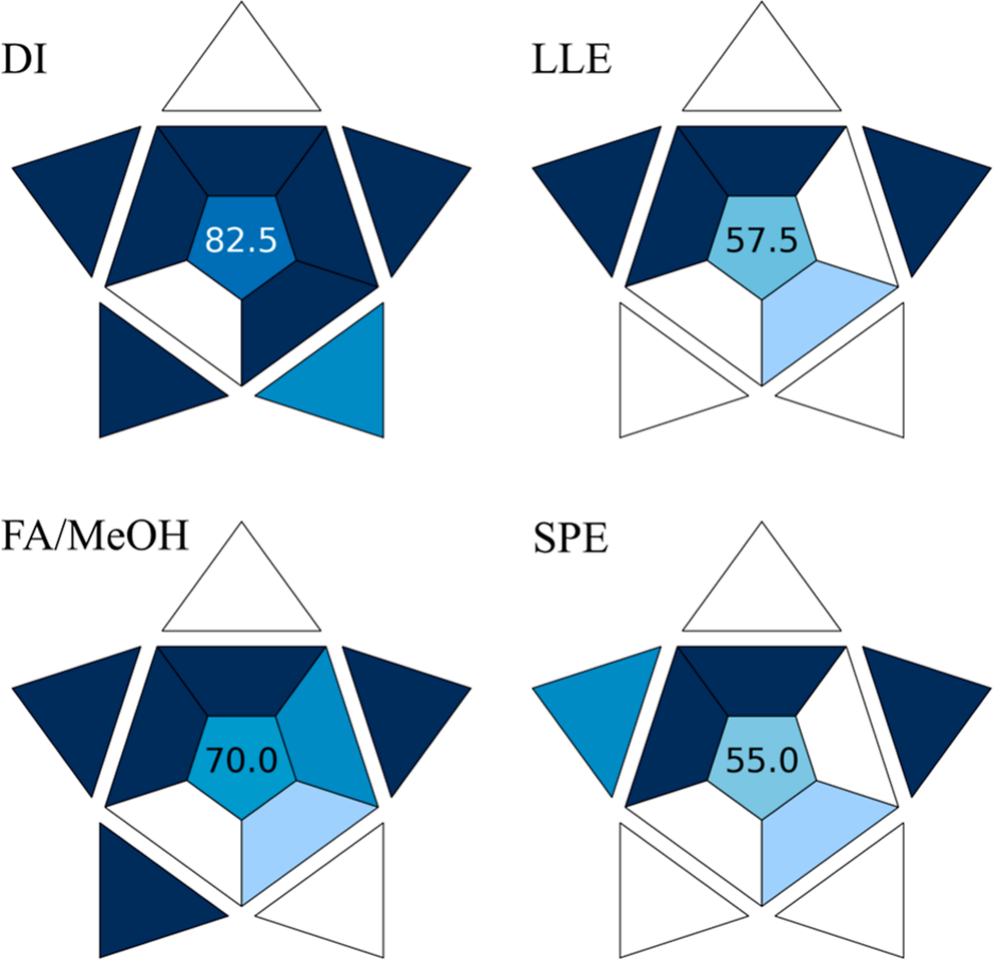
Comparison of the environmental impact for the DI-LC-MS lipidomic method vs standard lipid extraction methods, as assessed by the BAGI algorithm.

The DI-LC-MS method achieved the highest BAGI score (82.5) due to the absence of preconcentration, evaporation steps, additional reagents or solvents, and excessive use of disposable materials. In contrast, remaining procedures received lower scores, i.e., 57.5, 70.0, and 55.0 for the liquid–liquid extraction, single-phase extraction, and solid-phase extraction, respectively. While the BAGI algorithm identified the use of LC-Q-TOF-MS, a highly sophisticated analytical technique, and the analysis time required for chromatographic resolution of similar lipid species as potential drawbacks, we believe these aspects are essential for lipidomic analysis. For instance, high-resolution mass spectrometry provides crucial information, aiding analyte identification, and adequate resolution is necessary for accurate quantification. Therefore, these features, while potentially impacting the green credentials of the method, are unavoidable for this type of analysis. Hence, except for the limited lifespan of the chromatographic column, the developed approach can be considered a reliable and sustainable procedure for the lipidomic analysis of extracellular vesicles. Our novel approach seems to be particularly useful when the available samples are rather limited in volume and concentration.

## Discussion

The high interest in extracellular vesicles in the most varied disciplines has been accompanied by a demand for the in-depth characterization of their different biomolecules. If nucleic acid, protein, and even metabolite analyses have been extensively carried out, EV lipidomics is still lagging behind, mostly due to the amount of sample required. Traditionally, lipidomic analysis of EVs requires prior lipid extraction using techniques such as liquid–liquid extraction or single-phase extraction.^7,11–16^ These workflows isolate lipids and remove proteins and other potentially interfering molecules that can cause technical issues, including column clogging, reduced chromatographic resolution, decreased column lifespan, and signal suppression in mass spectrometry. However, these methods are often time-consuming, require high amounts of usually limited samples, use hazardous solvents, and can limit throughput and sustainability.

In this work, we demonstrated for the first time that the EV lipidome can be successfully analyzed by using a DI-LC-MS approach without prior extraction. Unlike traditional workflows, the DI-LC-MS method disrupts intact EVs directly within the chromatographic system under high organic solvent conditions. This simplifies EV lipidome analysis while maintaining the analytical performance. While previous studies have demonstrated the feasibility of direct injection of small-volume biological samples, even such as single cells for metabolomic/lipidomic analysis,^20,21^ this is the first report presenting a nonextraction method specifically applied to the lipidomics of EV. One of the advantages of this method is that it requires neither sophisticated equipment nor additional sample preparation step, such as disruption of the cell membrane. The reverse-phase mode was chosen for chromatographic separation of EV lipids, as it allows the separation of isobaric and isomeric lipid species. High content of the organic solvents used in the mobile phase, with starting composition 80% of the component A (5 mM ammonium formate in water/methanol 1/4; *v/v*) and 20% of the component B (5 mM ammonium formate in 2-propanol), enabled EV disintegration directly in the chromatographic column. While no selective extraction was performed, the chromatographic conditions were set up for nontargeted analysis of semi- to highly lipophilic compounds, ensuring that nonlipid components such as polar metabolites have no retention and therefore do not interfere with lipid separation.

Comparison of the DI-LC-MS approach with extraction-based methods (LLE, SPE, and single-phase extraction) shows that they can differ in terms of the lipidome coverage and unique features detected, in percentage distribution of lipids, as well as precision. We did not observe CE, DG, and TG in the SPE extract which is related to the selectivity of the stationary phase (Hybrid-SPE phospholipids) that only retains compounds containing phosphate moieties.^27^ A higher number of LPCs and their ether analogs were detected in the lipid extracts in comparison to the DI-LC-MS, which can indicate degradation of PCs and their ether analogs during the extraction step. On the other hand, the comparison of the percentage distribution calculated based on the lipids detected by all the methods shows that the lipid profiles of SM, CE, and PC are similar across all of the tested methods. Notably, the worst precision was obtained with the single-phase extraction, which can be connected with the low methanol solubility of nonpolar lipids and their precipitation in polar solvents, resulting in lipid loss, since it was shown that the recovery of lipids depends on the solvent used for the extraction step.^30^ The precision of the DI-LC-MS method was high, with all of the identified lipids characterized with a peak area CV lower than 20%. Similar precision was also observed for SPE and LLE. Since the focus of this study was to evaluate the differences between the DI-LC-MS and extraction-based workflows rather than to perform absolute lipid quantification, internal standards were not included in this part of the study. Instead, the precision of the method was assessed based on the peak area variation across replicates.

It has to be noted that for the lipidomic analysis of extracts and the DI-LC-MS, different chromatographic conditions were applied, a factor that could impact the obtained lipid profiles. The use of a high content of 2-propanol in the mobile phase in the DI-LC-MS approach was necessary for disrupting the EV membranes. Because of the difference in the LC–MS conditions and EV particle number, we used the percent relative amounts of the lipid species for comparison of the lipid profiles. This approach allowed us to eliminate differences resulting from the varying numbers of EV particles used and the different methods employed. The differences in lipidome coverage observed between extraction-based methods and DI-LC-MS likely result from the fundamental differences in how these methods process lipids. The direct release of lipids into the column in the DI-LC-MS method preserves lipids that might otherwise be lost or altered during extraction steps, affecting lipidome coverage.

Compared with the traditional extraction-based workflows, DI-LC-MS offers additional advantages. The DI-LC-MS approach requires minimal sample volumes (0.1–5 μL, depending on EV particle concentration), addressing a critical limitation in EV research where sample availability is often restricted. In this study, the sample volume was adjusted to prevent MS signal saturation. However, it can be easily adjusted and increased to aid the detection of less abundant species if required. Also, the chemical background was significantly reduced in the DI-LC-MS approach in comparison to that of lipid extraction. The DI-LC-MS eliminates the need for an enrichment step while reducing sample manipulation, simplifying the workflow, and minimizing contamination risks. These advantages make the method particularly attractive for applications involving highly limited or low-abundant samples, such as EVs obtained from patient-derived cells or limited amounts of specific biological fluids, such as liquid biopsies. Importantly, the DI-LC-MS aligns with the principles of green analytical chemistry by eliminating hazardous solvents such as chloroform and significantly reducing waste generation. These attributes make DI-LC-MS an environmentally sustainable alternative to the traditional extraction-based methods, particularly for high-throughput workflows.

The versatility of DI-LC-MS was demonstrated across mammalian and bacterial EV sources, including human plasma, fetal bovine serum, and cyanobacterial cells. The lipid profiles of CEV containing mainly MGDG, DGDG, and SQDG align with our prior studies on cyanobacterial membranes.^28^ Notably, MGDG 34:3, SQDG 32:0, and DGDG 34:3 were the most abundant species within their respective classes, both in cyanobacterial cells and in their EVs. Nevertheless, the relative distribution of the lipid species within lipid classes in CEVs varied from the previously reported lipid profiles of cyanobacterial cells.^28^ These variations could arise from differences in membrane remodeling during EV formation and/or cultivation conditions, which significantly impact the cyanobacterial lipidome. Additionally, cyanobacteria are composed of three distinct sets of membranes: thylakoids, the photosynthetic membranes, and the inner and outer membranes. For all of these reasons, direct comparisons between cyanobacterial EV and whole-cell lipidomes should be interpreted with caution. Similarly, the lipid species detected in human plasma EVs using DI-LC-MS are consistent with those reported in the previous studies employing conventional extraction-based techniques.^16,31,32^ Although previous lipidomic studies of human plasma EVs have reported a greater number of the identified lipid species, the primary objective of this study was not to achieve the deepest lipidome coverage but rather to evaluate and compare different lipid extraction and nonextraction workflows. In agreement, this study did not aim to capture the full EV lipidome, and so, future studies may need to adjust the DI-LC-MS workflow to expand lipid class coverage or the detection of a specific lipid class. Despite detecting fewer total lipids, DI-LC-MS effectively differentiated HPEV and FBSEV lipidomes, revealing significant variations in the lipid species composition and their relative abundance, for example, the enrichment in the LC-PUFA-containing PCs levels and LPCs in FBSEV in comparison to HPEV. These findings demonstrate the feasibility of the DI-LC-MS approach for comparative EV lipidomics while simultaneously underscoring the biological variability between different EV sources. It should be mentioned that partial coisolation of lipoproteins occurred in the fetal bovine serum EV samples, as confirmed by Western blot and TEM analysis. This may have influenced the lipid profile of FBSEVs, including the detection of CE and TG, which are lipid classes commonly associated with serum- and plasma-derived lipoproteins. Notably, CE and TG were also detected in the commercially obtained human plasma EVs, which were commercialized as an exosome sample. This further illustrates the difficulty in fully eliminating lipoprotein contamination in such sample types.^33^ Importantly, the primary objective of our study was to compare the analytical workflows and demonstrate that the DI-LC-MS method can detect meaningful differences in lipid profiles across various types of EVs. Since all the samples were processed using the same protocols and conditions, any non-EV particle contribution would equally affect each workflow. Therefore, we believe that the presence of non-EV components does not compromise the validity of the comparative conclusions drawn from this analysis.

While the DI-LC-MS method offers substantial advantages, some limitations were also detected, which are not present in the extraction-based methods. The primary drawback was a reduction in the column lifespan, accompanied by changes in peak shape (e.g., widening, tailing, and loss of symmetry). These changes were likely due to the adsorption of certain matrix components onto the stationary phase; however, they did not affect the precision of the method. Similarly, a decrease in the MS signal intensity was observed during the initial injections, potentially due to matrix-related contamination at the MS inlet. However, this can be easily overcome through regular LC–MS system equilibration, implementation of quality control samples, internal standards, and randomization of sample injection order.^34^ Still, the obtained MS signal intensity was high enough to obtain high lipidome coverage and appropriate precision in comparison to the conventional lipidomic methods using a priori extraction. While the column remained functional for approximately 30 injections, we observed gradual deterioration in the peak shape, likely due to the accumulation of matrix components. This suggests that the method is most appropriate for small-scale or short-term EV lipidomics studies where sample availability is typically limited.

This study demonstrated that each of the lipidomics workflows, namely, DI-LC-MS, LLE, single-phase extraction, and SPE, has unique strengths and limitations. Extraction-based workflows employ time-consuming and multistep procedures, which usually do not align with green chemistry principles. LLE provided broader lipidome coverage in comparison to the DI-LC-MS approach at the cost of greater sample and solvent demands. SPE, on the other hand, enabled selective extraction and enrichment of phospholipids, a feature that may be beneficial for studies requiring the detection of low-abundant GP or SP lipid species. Single-phase extraction, although with a simpler workflow, is solvent-sensitive for lipidome recovery and was characterized with the lowest precision. To this end, the novel DI-LC-MS approach offers simplicity and sustainability, making it an ideal choice for high-throughput studies or applications involving limited samples, such as EVs.

## Conclusions

This study demonstrated the applicability of DI-LC-MS for untargeted lipidomic analysis of extracellular vesicles and compared its performance to traditional extraction-based methods. Unlike conventional workflows requiring lipid extraction or protein removal, DI-LC-MS simplifies sample preparation by directly disrupting intact EVs in the chromatographic system under high organic solvent conditions. This approach significantly reduces sample processing time, minimizes losses of this precious material, eliminates the need for hazardous solvents, such as chloroform, and provides a green and efficient alternative to extraction-based lipidomics. While DI-LC-MS does not aim to replace extraction-based approaches, it offers a complementary, streamlined workflow that is particularly useful for studies prioritizing sample preservation and rapid analysis. By comparing different analytical strategies, this study provides a practical reference for researchers selecting lipidomics workflows suited to their experimental needs.

## Supplementary Material



## Data Availability

The lipidomics raw data have been deposited to the Bridge of Knowledge (https://mostwiedzy.pl/pl/open-research-data/catalog) with the data set identifier titled “Lipidomics raw data obtained by the DI-LC-MS and extraction-based workflows” under the link: https://mostwiedzy.pl/pl/open-research-data/lipidomics-raw-data-obtained-by-the-di-lc-ms-and-extraction-based-workflows,20312370121553-0?_share=3e6922736a93afcb with the data set identifier DOI: 10.34808/rqs6-pa04.

## Notes

Materials and methods used for the isolation of EV samples; characteristics of tested FBSEV samples; characteristics of tested CEV samples; lipids identified in CEV samples with their percentage distribution within lipid classes obtained using DI-LC-MS and extraction techniques; lipids identified in FBSEV samples with their percentage distribution within lipid classes obtained using DI-LC-MS and extraction techniques; lipids identified in HPEV samples with their percentage distribution within lipid classes obtained using DI-LC-MS and extraction techniques; a list of statistically significant lipid differences between HPEV and FBSEV; comparison of the number of detected MFs and precision of the DI-LC-MS method in untargeted analysis; a performance evaluation table, including the CV of peak area, width, and symmetry in DI-LC-MS; the impact of sample volume on the number of detected MFs in FBSEV extracts obtained by LLE, SPE, and single-phase extraction; column backpressure trace; lipid profiles of FBSEV obtained by LC–MS analysis of LLE extracts; lipid profiles of FBSEV obtained by LC–MS analysis of single-phase extracts; lipid profiles of FBSEV obtained by LC–MS analysis of SPE; and the percent relative amount calculated only for lipids detected by all methods and used for the radar plots (PDF)
