# Supplementary material for: Evaluating Novel Direct Injection Liquid Chromatography–Mass Spectrometry Method and Extraction-Based Workflows for Untargeted Lipidomics of Extracellular Vesicles
Source: J Proteome Res. 2025 Aug 12;24(11):5412–26. doi: 10.1021/acs.jproteome.5c00156 (PMC12604029; doi:10.1021/acs.jproteome.5c00156)
Supplement: Supplementary file 1 [file pr5c00156_si_001.pdf]

Supporting Information

Evaluating novel Direct Injection Liquid Chromatography-Mass Spectrometry (DI-LC-MS) method and Extraction-Based Workflows for Untargeted Lipidomics of Extracellular Vesicles

*Michał Młynarczyk[a], Felicja Gajdowska[b], Jorge Matinha-Cardoso[c,d,e], Paulo Oliveira[d,f], Paula Tamagnini[e,f], Mariusz Belka[g], Jagoda Mantej[b], Danuta Gutowska-Owsiak[h], Weronika Hewelt-Belka[a]**

[a] Department of Analytical Chemistry, Faculty of Chemistry, Gdańsk University of Technology, 80-233 Gdańsk, Poland

[b] Laboratory of Experimental and Translational Allergology and Pneumology, Medical University of Gdańsk, 80-214 Gdańsk, Poland

[c] MCbiology Doctoral Program, ICBAS – School of Medicine and Biomedical Sciences Abel Salazar, University of Porto, 4050-313 Porto, Portugal

[d] CIIMAR – Interdisciplinary Centre of Marine and Environmental Research, University of Porto, 4450-208 Matosinhos, Portugal

[e] i3S - Instituto de Investigação e Inovação em Saúde, University of Porto, 4200-135 Porto, Portugal

[f] Department of Biology, Faculty of Sciences, University of Porto, 4169-007 Porto, Portugal

[g] Department of Pharmaceutical Chemistry, Medical University of Gdańsk, 80-416 Gdańsk, Poland

[h] Laboratory of Experimental and Translational Immunology, Intercollegiate Faculty of Biotechnology of University of Gdańsk and Medical University of Gdańsk, University of Gdańsk, 80-307 Gdańsk, Poland

Table of contents

Materials and methods	p. S1-S4
Figure S1	p. S5
Figure S2	p. S6
Figure S3	p. S7
Figure S4	p.S7
Table S1	p. S8
Table S2	p. S10
Table S3	p. S15
Table S4	p. S20
Table S5	p. S21
Table S6	p. S22
Table S7	p. S23
Table S8	p. S28
Table S9	p. S33
Table S10	p. S41

Materials and methods

Reagents for fetal bovine serum EVs isolation and characterization.

The RPMI-1640, DMEM medium and Fetal Bovine Serum were purchased from Sigma-Aldrich (St. Louis, MO, USA). PBS Tablets were purchased from Thermo Fisher Scientific (Waltham, Massachusetts, USA). Stericup 0.1 μm was purchased from Millipore Sigma (Burlington, Massachusetts, USA). Ultracentrifuge tubes were purchased from Beckman Coulter (Brea, California, USA).

Synechocystis sp. PCC6803 cultivation

The cyanobacterium *Synechocystis* sp. PCC6803 (substrain GT-Kazusa; glucose tolerant, with S-layer and non-motile) was maintained in liquid BG11 medium in 100 mL Erlenmeyer flasks, on an orbital shaker (100 rpm), under a 16 h light (30–40 μmol/m^2/s)/8 h dark regimen, at 28 °C. Growth was monitored spectrophotometrically by analyzing the OD_{730}. Routinely, cyanobacterial strains were streaked on solid BG11 medium plates to assess the axenic state of

the cultures. Cells were initially cultivated in BG11 and sparged with air (1.2 L/min) in 500 mL glass gas washing bottles, in a final volume of 200 mL of culture. Cultivation was carried out at 30 °C with a 16 h light (40-50 μmol/m^2/s)/8h dark regimen, until cell density reached an OD_{730} of ≈ 1.0-1.2. This was later used as inoculum for a 3.5 L culture, cultivated in a 5 L bioreactor with an initial OD_{730} of 0.1. Cultivation was carried out at 25 °C with magnetic stirring (150 rpm), under a 16 h light (30 μmol/m^2/s)/8 h dark regimen, until an OD_{730} of ≈ 2. Then, biomass was separated from the extracellular medium by centrifugation (5000 *g*, for 15 min, at room temperature), followed by filtration through 0.45 μm pore size filters.

NTA of FBS EV samples

Nanoparticle tracking analysis (NTA) was used to determine the size and concentration of the isolated EVs. NTA was performed with NanoSight NS300 (Malvern Panalytical), equipped with a 488 nm laser, and an sCMOS camera. Camera level was set at 15, and the data was analysed with NanoSight NTA 3.4 software. EVs samples were thawed and diluted 3000-fold for FBS-derived EVs in PBS (filtered by 0.1 μm size filter). Videos of the samples were recorded 3-5 times for 60 seconds each. The background was measured with the same PBS as was used for the dilution of the samples.

TEM visualization

Negative-staining transmission electron microscopy was used to visualize EV samples. Five to 10 µL of sample were applied on the surface of formvar/carbon film coated mesh nickel grids (Electron Microscopy Sciences) and left standing for 2 min. Filter paper was used to remove liquid in excess, and 10µL of 1% uranyl acetate was added on to the grids and left standing for 10 s, after which liquid in excess was again removed with filter paper. Visualization was carried out on a Jeol JEM-1400 transmission electron microscope at 80 kV.

Western blot

4x Bolt™ LDS Sample Buffer (Thermo Fisher Scientific, Waltham, MA, USA) (4x diluted) was added to samples containing 50K of EVs, and the samples were heated for 10 min at 80°C. Samples were run on Bolt™ 4%–12% Bis-Tris Plus Gels (Thermo Fisher Scientific, Waltham, MA, USA) for 50 min using PowerEase™ 150V Power Supply (Thermo Fisher Scientific, Waltham, MA, USA). Proteins were transferred onto nitrocellulose membranes (iBlot™ 2 Transfer Stacks, nitrocellulose, regular size, Thermo Fisher Scientific, Waltham, MA, USA) using iBlot transfer system (iBlot 2 Dry Blotting System, Thermo Fisher Scientific, Waltham, MA, USA) and the membranes were blocked in 5% skimmed milk in TBS1X with 0,05% of Tween 20 (TBS-T) for one hour. Primary antibody incubations (diluted 1:250–1:1000) with 2,5% of BSA in TBS-T buffer were carried out at 4°C on a shaker overnight, and secondary antibody IRDye® 800CW (LI-COR Biosciences, Lincoln, NE, USA) in 1% of BSA in TBS-T buffer (dilution 1:25,000) for 45 min at RT. Membranes were scanned and analysed using Odyssey Clx Imaging System (LI-COR Biosciences, Lincoln, NE, USA). The following primary antibodies were used: mouse anti-CD63 (sc-5275, 1:1000, Santa Cruz Biotechnology, USA), mouse anti-flotillin-1 (610820, 1:250, BD Biosciences, USA), and mouse anti-apolipoprotein A-I (apoA-I; sc-376818, 1:250, Santa Cruz Biotechnology, USA). The secondary antibody was donkey anti-mouse IRDye® 800CW (926-32212, LI-COR Biosciences, USA).

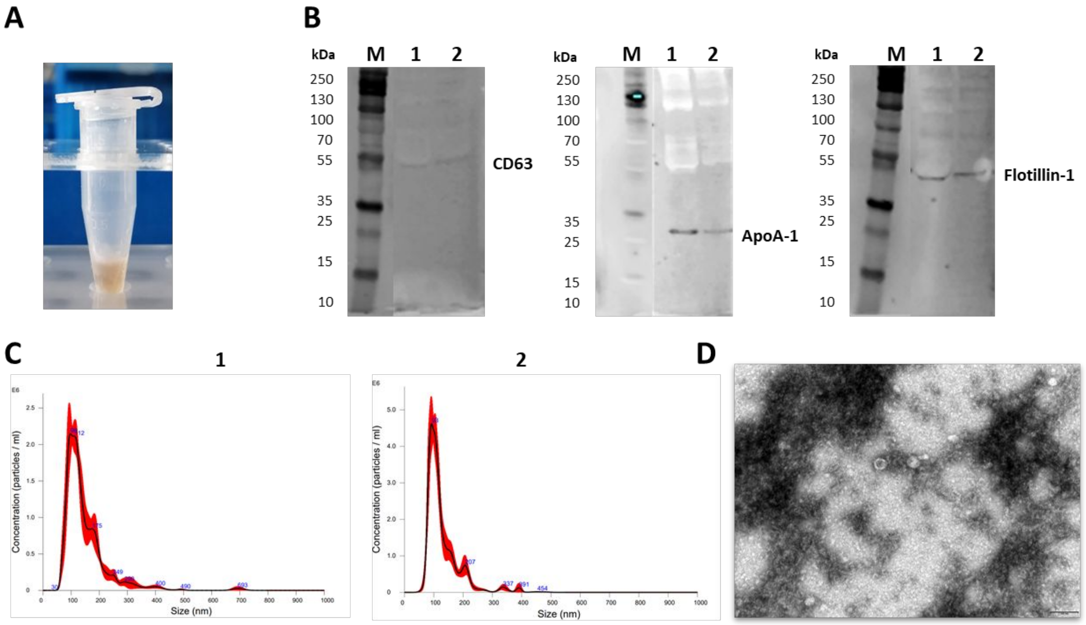

Figure S1. Extracellular vesicles (EVs) isolated from FBS used in the present study. (A) Aspect of the solution after suspending the FBS EVs pellet resulting from ultracentrifugation. (B) Identification of exosomal markers and lipoproteins in EVs sample (50K) by Western blot; 1- FBS-derived EVs mixed 1:1 with RPMI 1640; 2- FBS-derived EVs mixed 1:1 with DMEM; M - protein marker. Molecular masses are shown in kDa. (C) FBS EVs size distribution as analyzed by nanoparticle tracking analysis; 1- FBS-derived EVs mixed 1:1 with RPMI 1640; 2- FBS-derived EVs mixed 1:1 with DMEM. (D) Representative transmission electron micrographs of negatively stained FBS EVs magnified 100000x (scale bar: 100 nm).

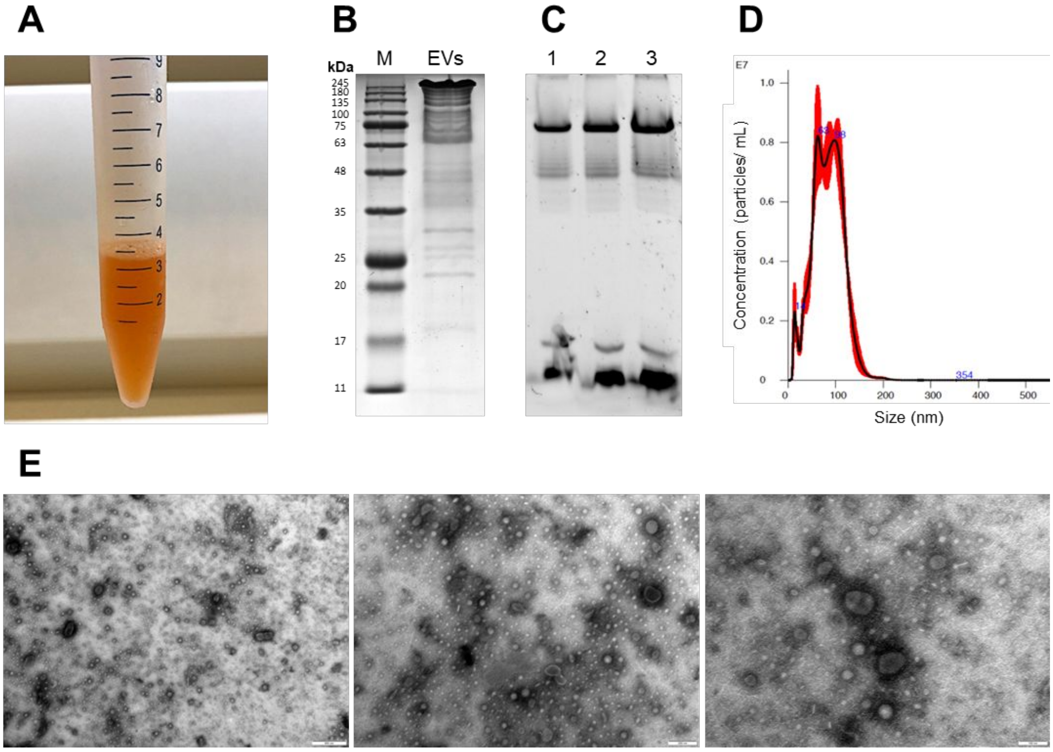

Figure S2. Extracellular vesicles isolated from the cyanobacterium *Synechocystis* sp. PCC 6803 used in the present study. (A) Aspect of the solution after suspending the cyanobacterial EV pellet resulting from ultracentrifugation. (B) Coomassie stained, 16% (w/v) SDS-polyacrylamide gel showing the cyanobacterial EV protein profile (EVs); M - protein marker. Molecular masses are shown in kDa. (C) Lipopolysaccharides (LPS) of the cyanobacterial EV preparation were detected after separating 12, 14 and 20 µg of LPS (lanes 1 to 3, respectively) by electrophoresis on a 16% (w/v) SDS-polyacrylamide gel, and staining with the Pro-Q™ Emerald 300 Lipopolysaccharide gel staining kit (Thermo Fisher Scientific). Typical low and high-molecular-weight LPS, corresponding to rough- and smooth-LPS forms, respectively, are detectable. (D) Cyanobacterial EV size distribution as analyzed by nanoparticle tracking analysis. (E) Transmission electron micrographs of negatively stained cyanobacterial EVs magnified 25000 x (panel on the left-hand side; scale bar: 500 nm), 50000 x (central panel; scale bar: 200 nm), and 100000 x (panel on the right-hand side: scale bar 100 nm).

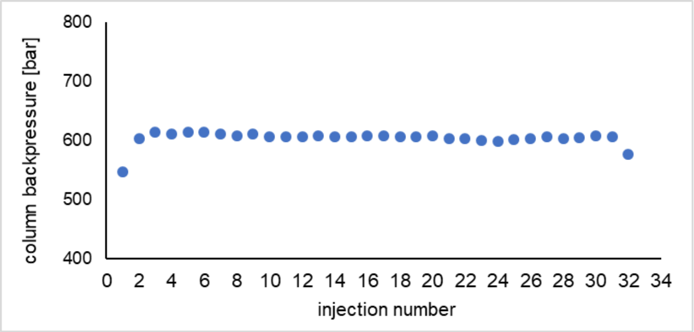

Figure S3. Column backpressure trace recorded over 30 consecutive injections of the CEV sample (injections 2–31). The first and last injections (injections 1 and 32) correspond to a reference sample containing lipid standards.

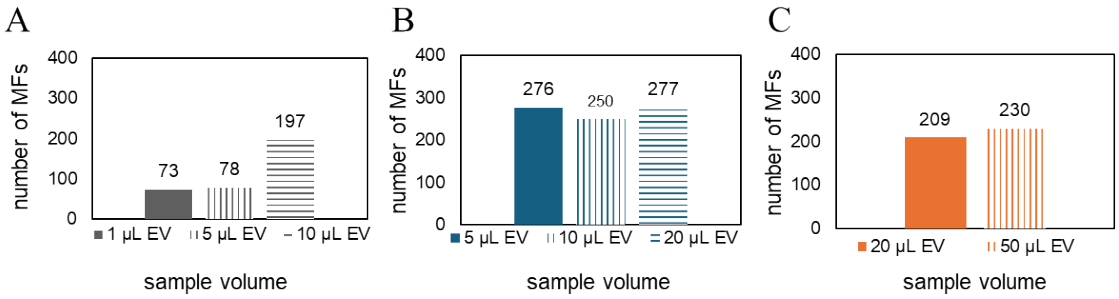

Figure S4. Number of MFs detected in extracts obtained from different volumes of fetal bovine EV sample with the use single phase extraction (methanol with 1% formic acid) (A), liquid-liquid extraction (B) and solid-phase extraction (C) techniques. Chromatographic condition 2 was applied.

Table S1. Characteristics of the identified lipids along with their percentage distribution within a class in CEV sample obtained by the DI-LC-MS method (chromatographic condition 1).

				% relative amount						
Name	Formula	Mass	RT	DI CEV_1	DI CEV_2	DI CEV_3	DI CEV_4	DI CEV_5	average relative amount	%CV of peak area
DGDG 32:0	C47 H88 O15	892.6123	8.29	11.0%	10.3%	10.9%	11.0%	10.6%	10.8%	5.0%
DGDG 32:1	C47 H86 O15	890.5973	6.76	7.6%	8.0%	7.8%	7.8%	7.7%	7.8%	4.2%
DGDG 34:1	C49 H90 O15	918.6312	8.89	13.2%	14.5%	14.1%	14.5%	13.9%	14.0%	4.1%
DGDG 34:2	C49 H88 O15	916.6125	7.49	21.5%	21.0%	20.0%	20.8%	21.7%	21.0%	5.9%
DGDG 34:3	C49 H86 O15	914.597	6.54	46.7%	46.2%	47.2%	45.9%	46.1%	46.4%	4.7%
total peak area DGDG				3002557.00	2837146.00	2822032.00	2783349.00	3118314.00		4.4%
MGDG 32:0	C41 H78 O10	730.5589	10.47	4.4%	4.4%	4.4%	4.4%	4.5%	4.4%	5.3%
MGDG 32:1	C41 H76 O10	728.5455	8.65	9.4%	9.5%	9.3%	9.6%	9.7%	9.5%	5.3%
MGDG 32:2	C41 H74 O10	726.5313	7.35	0.6%	0.6%	0.5%	0.6%	0.5%	0.6%	10.5%
MGDG 33:1	C42 H78 O10	742.5589	9.87	0.8%	0.8%	0.9%	0.9%	0.8%	0.8%	4.0%
MGDG 33:2	C42 H76 O10	740.5427	8.4	0.6%	0.6%	0.6%	0.6%	0.6%	0.6%	4.0%
MGDG 33:3	C42 H74 O10	738.5299	7.34	0.3%	0.3%	0.3%	0.3%	0.3%	0.3%	7.8%
MGDG 34:1	C43 H80 O10	756.5759	11.18	17.5%	17.4%	16.9%	17.6%	17.7%	17.4%	6.2%

MGDG 34:2	C43 H78 O10	754.5635	9.55	21.1%	20.6%	20.7%	20.7%	20.8%	20.8%	6.1%
MGDG 34:3	C43 H76 O10	752.5452	8.4	43.8%	44.1%	44.7%	43.8%	43.4%	44.0%	5.0%
MGDG 34:4 iso 1	C43 H74 O10	750.528	6.81	1.0%	1.0%	1.0%	1.1%	1.1%	1.0%	3.5%
MGDG 34:4 iso 2	C43 H74 O10	750.5274	7.18	0.6%	0.6%	0.6%	0.6%	0.6%	0.6%	5.3%
total peak area SQDG				80358119	73299581	76691708	77866384	85999850		5.4%
SQDG 32:0	C41 H78 O12 S	794.5242	4.96	65.1%	64.6%	64.0%	63.7%	62.5%	64.0%	6.8%
SQDG 32:1	C41 H76 O12 S	792.507	4.01	13.0%	13.4%	13.8%	13.9%	15.0%	13.8%	1.8%
SQDG 33:0	C42 H80 O12 S	808.5379	5.75	0.3%	0.3%	0.3%	0.3%	0.3%	0.3%	5.4%
SQDG 33:1	C42 H78 O12 S	806.5231	4.63	0.8%	0.9%	0.9%	0.8%	0.9%	0.8%	5.7%
SQDG 34:0	C43 H82 O12 S	822.5522	6.64	0.5%	0.5%	0.5%	0.5%	0.5%	0.5%	3.5%
SQDG 34:1	C43 H80 O12 S	820.5381	5.36	10.1%	10.0%	10.0%	10.2%	10.2%	10.1%	5.0%
SQDG 34:2	C43 H78 O12 S	818.5216	4.47	7.6%	7.6%	7.8%	7.8%	7.8%	7.7%	4.5%
SQDG 34:3	C43 H76 O12 S	816.5031	4.96	2.7%	2.7%	2.7%	2.8%	2.9%	2.7%	3.2%
Total peak area				13841506	13610537	12889723	12384014	14486788		5.5%

Table S2. Characteristics of the identified lipids along with their percentage distribution within a class in FBSEV sample obtained by the DI-LC-MS method (chromatographic condition 1).

				% relative amount						
Name	Formula	Mass	RT	DI FBSEV_1	DI FBSEV_2	DI FBSEV_3	DI FBSEV_4	DI FBSEV_5	average relative amount	%CV of peak area
CE 14:0	C41 H72 O2	596.55	33.7	0.7%	0.8%	0.7%	0.7%	0.7%	0.7%	7.7%
CE 16:0	C43 H76 O2	624.5842	36.7	7.7%	7.6%	7.4%	7.2%	6.8%	7.3%	9.2%
CE 16:1	C43 H74 O2	622.5684	34.5	8.1%	7.9%	7.9%	7.2%	7.5%	7.7%	8.2%
CE 18:0	C45 H80 O2	652.6141	39.4	0.6%	0.6%	0.6%	0.6%	0.6%	0.6%	7.4%
CE 18:1	C45 H78 O2	650.6004	37.3	24.6%	24.6%	24.8%	25.0%	25.0%	24.8%	7.1%
CE 18:2	C45 H76 O2	648.585	35.4	20.0%	20.6%	20.3%	20.8%	21.0%	20.5%	7.4%
CE 18:3	C45 H74 O2	646.5683	33.7	2.8%	2.7%	2.7%	2.7%	2.8%	2.8%	5.5%
CE 20:3	C47 H78 O2	674.5996	36.4	3.3%	3.1%	3.3%	2.8%	3.2%	3.1%	7.5%
CE 20:4	C47 H76 O2	672.5845	34.9	20.3%	20.0%	20.1%	20.5%	20.0%	20.2%	7.3%
CE 20:5	C47 H74 O2	670.5678	33.2	5.0%	5.1%	5.0%	5.2%	5.1%	5.1%	7.6%
CE 22:6	C49 H76 O2	696.5839	34.5	6.5%	6.7%	6.7%	6.8%	6.8%	6.7%	7.7%
cholesterol	C27 H46 O	386.3559	6.3	0.4%	0.4%	0.4%	0.5%	0.5%	0.4%	8.2%
total peak area CE				38640235.00	45476595.00	44544396.00	43946573.00	38459963.00		7.2%

LPC 14:0	C22 H46 N O7 P	467.3033	1.2	0.16%	0.16%	0.16%	0.15%	0.16%	0.16%	5.9%
LPC 16:0	C24 H50 N O7 P	495.3332	1.4	7.88%	7.81%	7.68%	7.69%	7.69%	7.75%	5.5%
LPC 16:1	C24 H48 N O7 P	493.317	1.2	0.46%	0.44%	0.45%	0.45%	0.45%	0.45%	5.3%
LPC 18:0	C26 H54 N O7 P	523.3644	1.8	7.09%	7.01%	7.22%	6.99%	7.28%	7.12%	5.9%
LPC 18:1	C26 H52 N O7 P	521.3487	1.4	4.87%	4.81%	4.75%	4.76%	4.82%	4.80%	5.3%
LPC 20:1	C28 H56 N O7 P	549.3814	1.9	0.21%	0.22%	0.21%	0.22%	0.22%	0.22%	8.1%
LPC 20:3	C28 H52 N O7 P	545.3461	1.8	1.05%	1.38%	1.45%	1.41%	1.32%	1.32%	15.9%
LPC 22:5	C30 H52 N O7 P	569.3482	1.4	0.63%	0.64%	0.59%	0.63%	0.63%	0.63%	5.7%
PC 30:0	C38 H76 N O8 P	705.5303	6.7	0.37%	0.37%	0.38%	0.37%	0.36%	0.37%	7.5%
PC 31:0	C39 H78 N O8 P	719.5458	7.8	0.21%	0.21%	0.18%	0.20%	0.20%	0.20%	5.6%
PC 32:0	C40 H80 N O8 P	733.5624	8.9	3.44%	3.48%	3.43%	3.40%	3.43%	3.44%	6.1%
PC 32:1	C40 H78 N O8 P	731.5468	7.3	1.37%	1.30%	1.32%	1.35%	1.35%	1.34%	5.1%
PC 34:0	C42 H84 N O8 P	761.5925	11.5	1.50%	1.47%	1.45%	1.50%	1.48%	1.48%	5.7%
PC 34:1	C42 H82 N O8 P	759.5782	9.6	21.02%	20.82%	20.82%	20.62%	20.84%	20.82%	5.6%
PC 34:2	C42 H80 N O8 P	757.5626	8.1	1.36%	1.36%	1.36%	1.32%	1.35%	1.35%	5.9%
PC 35:1	C43 H84 N O8 P	773.5924	10.9	1.16%	1.17%	1.32%	1.32%	1.33%	1.26%	10.2%
PC 36:1	C44 H86 N O8 P	787.6102	12.3	14.72%	14.79%	14.80%	14.93%	14.84%	14.82%	6.3%

PC 36:2	C44 H84 N O8 P	785.5937	10.3	3.78%	3.65%	3.48%	3.71%	3.51%	3.62%	5.8%
PC 36:3	C44 H82 N O8 P	783.578	9.0	1.32%	1.23%	1.47%	1.41%	1.37%	1.36%	10.0%
PC 36:4	C44 H80 N O8 P	781.5625	8.2	1.78%	1.95%	1.86%	1.88%	1.85%	1.86%	8.3%
PC 36:5	C44 H78 N O8 P	779.5462	7.1	0.16%	0.13%	0.15%	0.14%	0.13%	0.14%	7.7%
PC 38:3	C46 H86 N O8 P	811.6092	11.7	3.40%	3.40%	3.38%	3.43%	3.41%	3.40%	6.1%
PC 38:4	C46 H84 N O8 P	809.594	10.8	5.72%	5.67%	5.65%	5.72%	5.68%	5.69%	5.9%
PC 38:5	C46 H82 N O8 P	807.5774	8.8	3.78%	3.71%	3.79%	3.67%	3.83%	3.76%	5.4%
PC 38:6	C46 H80 N O8 P	805.5617	8.2	2.06%	2.06%	2.02%	2.02%	1.98%	2.03%	6.2%
PC 38:7	C46 H78 N O8 P	803.5436	8.2	0.20%	0.20%	0.20%	0.20%	0.19%	0.20%	8.1%
PC 40:4	C48 H88 N O8 P	837.6237	12.9	0.34%	0.42%	0.36%	0.37%	0.36%	0.37%	11.4%
PC 40:5	C48 H86 N O8 P	835.6087	11.4	4.32%	4.33%	4.28%	4.36%	4.30%	4.32%	6.2%
PC 40:6	C48 H84 N O8 P	833.593	10.7	3.84%	3.88%	3.89%	3.90%	3.86%	3.88%	6.6%
PC 40:7	C48 H82 N O8 P	831.5748	10.8	0.51%	0.56%	0.54%	0.53%	0.49%	0.52%	10.0%
PC 40:8	C48 H80 N O8 P	829.5592	8.8	0.37%	0.38%	0.38%	0.36%	0.35%	0.37%	7.9%
PC 40:9	C48 H78 N O8 P	827.5432	8.2	0.21%	0.22%	0.22%	0.22%	0.21%	0.22%	8.4%
PC 42:8	C50 H84 N O8 P	857.5903	11.4	0.38%	0.39%	0.39%	0.40%	0.37%	0.39%	8.1%
PC 42:9	C50 H82 N O8 P	855.5746	10.7	0.36%	0.36%	0.36%	0.35%	0.35%	0.36%	6.3%

total peak area PC				108055631	122784263	126548863	125266460	113383706		6.1%
PC O-32:1	C40 H80 N O7 P	717.5673	10.3	12.0%	11.8%	11.9%	12.0%	11.4%	11.8%	5.7%
PC O-34:1	C42 H84 N O7 P	745.5977	11.2	44.0%	43.4%	43.6%	45.3%	44.9%	44.2%	5.5%
PC O-34:2	C42 H82 N O7 P	743.582	11.0	28.4%	29.0%	28.6%	28.1%	28.1%	28.4%	5.2%
PC O-36:5	C44 H80 N O7 P	765.5666	9.4	8.6%	8.6%	8.9%	7.5%	8.2%	8.3%	6.6%
PC O-38:5	C46 H84 N O7 P	793.5977	10.3	7.0%	7.3%	7.0%	7.2%	7.3%	7.2%	5.3%
total peak area PC-O				2581998	2838456	2920175	2967121	2710151		5.0%
SM 32:1; O2	C37 H75 N2 O6 P	674.5362	4.7	1.1%	1.0%	1.1%	1.0%	1.0%	1.0%	6.1%
SM 33:1; O2	C38 H77 N2 O6 P	688.5519	5.5	1.4%	1.4%	1.4%	1.4%	1.5%	1.4%	6.8%
SM 34:0; O2	C39 H81 N2 O6 P	704.5833	7.2	1.8%	1.7%	1.7%	1.8%	1.8%	1.8%	5.7%
SM 34:1; O2	C39 H79 N2 O6 P	702.5685	6.4	37.0%	36.7%	36.3%	36.3%	36.3%	36.5%	6.2%
SM 34:2; O2	C39 H77 N2 O6 P	700.552	5.2	3.4%	3.4%	3.4%	3.3%	3.4%	3.4%	6.1%
SM 35:1; O2	C40 H81 N2 O6 P	716.5829	7.5	1.0%	1.0%	1.0%	1.0%	1.0%	1.0%	7.1%
SM 36:1; O2	C41 H83 N2 O6 P	730.5992	8.6	6.5%	6.5%	6.6%	6.3%	6.6%	6.5%	6.3%
SM 36:2; O2	C41 H81 N2 O6 P	728.5831	7.1	2.2%	2.3%	2.3%	2.2%	2.3%	2.2%	7.1%
SM 38:1; O2	C43 H87 N2 O6 P	758.6297	11.2	1.6%	1.6%	1.6%	1.6%	1.7%	1.6%	5.5%
SM 38:2; O2	C43 H85 N2 O6 P	756.6136	9.5	0.5%	0.6%	0.5%	0.5%	0.5%	0.5%	17.4%

SM 39:1; O2	C44 H89 N2 O6 P	772.6448	12.7	1.0%	1.0%	1.0%	1.0%	1.0%	1.0%	4.1%
SM 40:1; O2	C45 H91 N2 O6 P	786.6616	14.1	10.5%	10.2%	10.3%	10.4%	10.4%	10.4%	6.3%
SM 40:2 iso 1; O2	C45 H89 N2 O6 P	784.6444	11.7	1.7%	1.9%	1.8%	2.0%	1.8%	1.8%	10.5%
SM 40:2 iso 2; O2	C45 H89 N2 O6 P	784.6455	12.2	2.6%	3.1%	2.7%	3.0%	2.7%	2.8%	12.3%
SM 41:1; O2	C46 H93 N2 O6 P	800.6762	15.6	2.0%	1.9%	1.9%	2.0%	1.8%	1.9%	7.8%
SM 41:2; O2	C46 H91 N2 O6 P	798.6605	13.0	1.6%	1.6%	1.7%	1.7%	1.6%	1.6%	8.8%
SM 42:1; O2	C47 H95 N2 O6 P	814.6926	17.1	6.1%	6.0%	6.4%	6.2%	6.3%	6.2%	7.7%
SM 42:2; O2	C47 H93 N2 O6 P	812.6783	14.4	13.5%	13.5%	13.7%	13.7%	13.7%	13.6%	7.0%
SM 42:3; O2	C47 H91 N2 O6 P	810.661	12.6	4.4%	4.5%	4.6%	4.7%	4.7%	4.6%	8.2%
Total peak area SM				25255175	28789861	29308807	29369624	26538563		6.7%

Table S3. Characteristics of the identified lipids along with their percentage distribution in HPEV sample obtained by the DI-LC-MS method (chromatographic condition 1).

				% relative amount						
Name	Formula	Mass	RT	DI HPEV_1	DI HPEV_2	DI HPEV_3	DI HPEV_4	DI HPEV_5	average relative amount	%CV of peak area
CE 14:0	C41 H72 O2	596.55	33.7	0.22%	0.25%	0.22%	0.22%	0.21%	0.22%	8.31%
CE 16:0	C43 H76 O2	624.5842	36.67	2.61%	2.77%	2.74%	2.73%	2.69%	2.71%	3.97%
CE 16:1	C43 H74 O2	622.5684	34.45	3.41%	3.53%	3.61%	3.55%	3.63%	3.54%	3.22%
CE 18:0	C45 H80 O2	652.6141	39.38	0.18%	0.19%	0.20%	0.20%	0.19%	0.19%	6.07%
CE 18:1	C45 H78 O2	650.6004	37.29	12.79%	13.18%	12.99%	12.98%	13.13%	13.01%	2.53%
CE 18:2	C45 H76 O2	648.585	35.43	72.99%	72.50%	72.90%	73.12%	72.55%	72.81%	2.20%
CE 18:3	C45 H74 O2	646.5683	33.69	1.37%	1.36%	1.37%	1.29%	1.36%	1.35%	2.74%
CE 20:3	C47 H78 O2	674.5996	36.38	0.51%	0.56%	0.47%	0.50%	0.53%	0.52%	6.34%
CE 20:4	C47 H76 O2	672.5845	34.86	5.06%	4.92%	4.79%	4.64%	4.97%	4.88%	2.37%
CE 20:5	C47 H74 O2	670.5678	33.19	0.22%	0.23%	0.19%	0.24%	0.21%	0.22%	9.08%
CE 22:6	C49 H76 O2	696.5839	34.53	0.30%	0.26%	0.28%	0.27%	0.26%	0.27%	5.60%
cholesterol	C27 H46 O	386.3559	6.3158	0.34%	0.26%	0.22%	0.26%	0.27%	0.27%	14.80%
total peak area CE				137734898	142086919	141611789	142200174	135909867		2.07%
DG 34:1	C37 H70 O5	594.521	15.43	41.53%	41.81%	41.60%	41.39%	42.71%	41.81%	3.84%

DG 34:2	C37 H68 O5	592.5072	13.51	58.47%	58.19%	58.40%	58.61%	57.29%	58.19%	4.64%
total peak area DG				6054876	6495710	6599909	6702510	6223593	6415320	4.19%
LPC 16:0	C24 H50 N O7 P	495.3332	1.3802	0.40%	0.50%	0.48%	0.50%	0.47%	0.47%	12.64%
LPC 18:0	C26 H54 N O7 P	523.3644	1.7518	0.87%	1.06%	1.02%	1.06%	0.98%	1.00%	11.49%
LPC 18:1	C26 H52 N O7 P	521.3487	1.4252	0.11%	0.12%	0.12%	0.12%	0.11%	0.11%	8.36%
LPC 20:3	C28 H52 N O7 P	545.3461	1.7518	0.67%	0.83%	0.82%	0.86%	0.79%	0.79%	12.59%
PC 30:0	C38 H76 N O8 P	705.5303	6.7356	0.36%	0.40%	0.38%	0.38%	0.38%	0.38%	6.70%
PC 31:0	C39 H78 N O8 P	719.5458	7.7815	0.06%	0.07%	0.06%	0.07%	0.06%	0.06%	6.26%
PC 32:0	C40 H80 N O8 P	733.5624	8.9374	2.28%	2.42%	2.39%	2.40%	2.40%	2.38%	6.24%
PC 32:1	C40 H78 N O8 P	731.5468	7.3337	1.53%	1.60%	1.55%	1.55%	1.54%	1.55%	5.94%
PC 34:0	C42 H84 N O8 P	761.5925	11.5376	0.42%	0.44%	0.43%	0.43%	0.43%	0.43%	5.89%
PC 34:1	C42 H82 N O8 P	759.5782	9.610727	25.83%	26.73%	26.23%	26.83%	26.55%	26.43%	5.55%
PC 34:2	C42 H80 N O8 P	757.5626	8.1228	39.92%	39.14%	40.30%	39.29%	39.54%	39.64%	5.60%
PC 35:1	C43 H84 N O8 P	773.5924	10.90227	0.28%	0.28%	0.27%	0.27%	0.28%	0.28%	3.25%
PC 36:1	C44 H86 N O8 P	787.6102	12.3072	4.58%	4.67%	4.61%	4.56%	4.64%	4.61%	5.04%
PC 36:2	C44 H84 N O8 P	785.5937	10.2997	1.66%	1.60%	1.65%	1.63%	1.65%	1.64%	4.84%
PC 36:3	C44 H82 N O8 P	783.578	9.0464	4.35%	4.14%	4.00%	3.98%	4.02%	4.10%	4.79%

PC 36:4	C44 H80 N O8 P	781.5625	8.2411	6.79%	6.45%	6.25%	6.58%	6.63%	6.54%	3.19%
PC 36:5	C44 H78 N O8 P	779.5462	7.0745	0.20%	0.19%	0.19%	0.19%	0.20%	0.20%	3.41%
PC 38:3	C46 H86 N O8 P	811.6092	11.7094	2.00%	2.04%	1.98%	1.91%	1.94%	1.97%	5.79%
PC 38:4	C46 H84 N O8 P	809.594	10.7731	4.19%	4.01%	3.99%	4.03%	4.02%	4.05%	4.49%
PC 38:5	C46 H82 N O8 P	807.5774	8.8039	0.58%	0.49%	0.56%	0.56%	0.58%	0.55%	6.52%
PC 38:6	C46 H80 N O8 P	805.5617	8.1677	0.85%	0.80%	0.78%	0.79%	0.79%	0.80%	4.17%
PC 38:7	C46 H78 N O8 P	803.5436	8.2414	0.74%	0.74%	0.72%	0.75%	0.76%	0.74%	4.27%
PC 40:4	C48 H88 N O8 P	837.6237	12.8588	0.14%	0.11%	0.13%	0.12%	0.13%	0.13%	7.16%
PC 40:5	C48 H86 N O8 P	835.6087	11.3884	0.15%	0.14%	0.13%	0.14%	0.14%	0.14%	3.79%
PC 40:6	C48 H84 N O8 P	833.593	10.6837	0.36%	0.34%	0.33%	0.34%	0.33%	0.34%	5.36%
PC 40:7	C48 H82 N O8 P	831.5748	10.7723	0.45%	0.43%	0.42%	0.43%	0.42%	0.43%	5.29%
PC 40:8	C48 H80 N O8 P	829.5592	8.805	0.06%	0.08%	0.06%	0.07%	0.07%	0.07%	11.68%
PC 40:9	C48 H78 N O8 P	827.5432	8.1597	0.11%	0.11%	0.11%	0.11%	0.10%	0.11%	7.49%
PC 42:8	C50 H84 N O8 P	857.5903	11.3861	0.02%	0.02%	0.02%	0.02%	0.02%	0.02%	5.19%
PC 42:9	C50 H82 N O8 P	855.5746	10.6836	0.04%	0.04%	0.04%	0.04%	0.04%	0.04%	4.38%
total peak area PC				303485253	317025328	335564016	323009540	294717272		5.11%
SM 32:1; O2	C37 H75 N2 O6 P	674.5362	4.7357	1.77%	2.00%	1.98%	1.95%	1.93%	1.93%	9.44%

SM 33:1; O2	C38 H77 N2 O6 P	688.5519	5.5225	1.09%	1.17%	1.14%	1.15%	1.15%	1.14%	7.20%
SM 34:0; O2	C39 H81 N2 O6 P	704.5833	7.2372	1.23%	1.29%	1.30%	1.25%	1.26%	1.27%	7.57%
SM 34:1; O2	C39 H79 N2 O6 P	702.5685	6.4219	43.94%	43.80%	44.40%	44.34%	43.78%	44.05%	6.17%
SM 34:2; O2	C39 H77 N2 O6 P	700.552	5.2229	3.27%	3.50%	3.41%	3.40%	3.44%	3.40%	6.84%
SM 35:1; O2	C40 H81 N2 O6 P	716.5829	7.4728	0.55%	0.54%	0.51%	0.55%	0.57%	0.54%	2.74%
SM 36:1; O2	C41 H83 N2 O6 P	730.5992	8.603	5.63%	5.86%	5.84%	5.89%	5.76%	5.79%	7.04%
SM 36:2; O2	C41 H81 N2 O6 P	728.5831	7.115	2.41%	2.43%	2.43%	2.42%	2.48%	2.43%	5.41%
SM 38:1; O2	C43 H87 N2 O6 P	758.6297	11.1898	1.66%	1.63%	1.60%	1.59%	1.64%	1.63%	4.34%
SM 38:2; O2	C43 H85 N2 O6 P	756.6136	9.4704	1.20%	1.19%	1.20%	1.20%	1.20%	1.20%	5.43%
SM 39:1; O2	C44 H89 N2 O6 P	772.6448	12.7484	0.88%	0.88%	0.84%	0.83%	0.82%	0.85%	6.15%
SM 40:1; O2	C45 H91 N2 O6 P	786.6616	14.064	7.18%	7.12%	7.14%	7.16%	7.19%	7.16%	5.37%
SM 40:2 iso 1; O2	C45 H89 N2 O6 P	784.6444	11.7184	1.16%	1.14%	1.16%	1.08%	1.06%	1.12%	8.36%
SM 40:2 iso 2; O2	C45 H89 N2 O6 P	784.6455	12.2054	3.33%	3.32%	3.06%	3.13%	3.29%	3.23%	3.75%
SM 41:1; O2	C46 H93 N2 O6 P	800.6762	15.5789	2.68%	2.67%	2.64%	2.62%	2.63%	2.65%	5.57%
SM 41:2; O2	C46 H91 N2 O6 P	798.6605	13.0493	0.64%	0.66%	0.65%	0.61%	0.63%	0.64%	7.42%
SM 42:1; O2	C47 H95 N2 O6 P	814.6926	17.1107	4.64%	4.41%	4.39%	4.49%	4.50%	4.49%	4.25%
SM 42:2; O2	C47 H93 N2 O6 P	812.6783	14.4338	10.85%	10.57%	10.50%	10.74%	10.85%	10.70%	4.33%

SM 42:3; O2	C47 H91 N2 O6 P	810.661	12.5713	5.66%	5.61%	5.56%	5.38%	5.60%	5.56%	5.23%
SM 43:2; O2	C48 H95 N2 O6 P	826.6915	15.3086	0.25%	0.21%	0.25%	0.21%	0.24%	0.23%	9.38%
total peak area SM				7053967	7470581	7836995	7171038	6866758		5.23%
PC O-32:1	C40 H80 N O7 P	717.5673	10.2852	16.23%	16.26%	16.05%	15.80%	15.79%	16.03%	5.94%
PC O-34:1	C42 H84 N O7 P	745.5977	11.1505	34.75%	36.17%	35.44%	34.85%	35.04%	35.25%	6.39%
PC O-34:2	C42 H82 N O7 P	743.582	10.9775	14.29%	13.90%	14.04%	13.41%	13.62%	13.85%	6.09%
PC O-36:5	C44 H80 N O7 P	765.5666	9.4391	23.04%	21.54%	21.96%	22.41%	23.05%	22.40%	3.46%
PC O-38:5	C46 H84 N O7 P	793.5977	10.3347	11.69%	12.13%	12.51%	13.52%	12.50%	12.47%	8.74%
total peak area PC-O				4980332	5301673	5487037	5285027	4783127		5.45%
TG 48:1	C51 H96 O6	804.7248	35.86	3.80%	4.64%	4.70%	4.64%	4.25%	4.40%	14.85%
TG 50:1	C53 H100 O6	832.7571	38.22	9.94%	11.52%	12.48%	12.29%	12.23%	11.69%	13.47%
TG 50:2 iso 1	C53 H98 O6	830.7374	36.5	8.09%	7.38%	7.63%	7.70%	7.98%	7.75%	4.09%
TG 50:2 iso 2	C53 H98 O6	830.7374	36.7	6.37%	8.01%	7.72%	7.14%	7.45%	7.34%	13.15%
TG 50:3	C53 H96 O6	828.7206	34.93	4.08%	4.04%	4.13%	4.03%	3.92%	4.04%	6.57%
TG 52:2	C55 H102 O6	858.7694	38.8	34.02%	33.08%	33.89%	33.31%	33.30%	33.52%	5.54%
TG 52:3	C55 H100 O6	856.7518	37.32	33.19%	30.90%	28.99%	30.41%	30.38%	30.77%	3.64%
TG 56:3	C59 H108 O6	912.8125	37.33	0.12%	0.10%	0.11%	0.12%	0.11%	0.11%	7.45%

TG 58:2	C61 H114 O6	942.8604	38.77	0.23%	0.20%	0.21%	0.21%	0.22%	0.21%	3.68%
TG 58:3	C61 H112 O6	940.8439	37.33	0.15%	0.15%	0.14%	0.15%	0.15%	0.15%	5.95%
total peak area TG				457339103	522270921	523137748	535803195	503275717		6.06%

Table S4. List of significantly different lipids between HPEV and FBSEV samples. Fold change was calculated using %relative amount of lipid specie in HPEV and FBSEV samples.

Lipid name	FC FBSEV/FC HPEV	P value
CE 14:0	3.25	4.903e-09
CE 16:0	2.709	4.6273e-06
CE 16:1	2.1788	5.3516e-06
CE 18:0	3.1042	2.3893e-11
CE 18:2	0.28166	2.5635e-15
CE 18:3	2.04	1.3893e-09
CE 20:3	6.0934	4.0611e-06
CE 20:4	4.139	5.9449e-13
CE 20:5	23.33	5.6865e-09
CE 22:6	24.467	6.042e-09
LPC 16:0	16.489	1.5895e-11
LPC 18:0	7.1323	1.4841e-11
LPC 18:1	41.397	1.8595e-09
PC 31:0	3.125	1.1257e-06
PC 34:0	3.4419	2.262e-09
PC 34:2	0.034058	5.3811e-09
PC 35:1	4.5652	1.3595e-05
PC 36:1	3.2125	4.4976e-14
PC 36:2	2.2137	2.2894e-06
PC 36:3	0.33187	1.4037e-08
PC 36:4	0.28502	1.3714e-07
PC 38:5	6.7798	2.5085e-11
PC 38:6	2.5287	9.0401e-12
PC 38:7	0.26685	1.5341e-08
PC 40:4	2.9365	1.0395e-05
PC 40:5	30.843	1.0135e-10
PC 40:6	11.394	1.4052e-13
PC 40:8	5.4118	1.4133e-09
PC 42:8	19.3	2.2574e-07
PC 42:9	8.9	2.1654e-08
PC O-34:2	2.0531	4.8832e-12
PC O-36:5	0.37321	6.6034e-10
SM 38:2; O2	0.43406	3.7928e-06
SM 41:2; O2	2.5705	2.3568e-07

Table S5. Comparison of number of detected MFs and precision for DI-LC-MS methods in untargeted approach

	FBSEV	HPEV	CEV
number of MFs	174	483	171
number MFs with %CV of peak volume <20%	141	300	122
% of total MFs with %CV of peak volume <20%	81%	62%	71%
total signal variation (%CV)	7%	7%	5%
number of identified lipids	70	79	24
% of lipids with %CV if peak area <20%	100%	100%	100%
injection volume (µL)	0.1	0.5	1
number of particles injected	6x10^7	1.5x10^9	1x10^9

Table S6. Variation of peak area, width and symmetry during the 30 following direct injections of EVs samples and reference sample containing lipid standards.

	Compound	Fold change (FC) of peak area in a reference sample** before inj. of CEV/after 9 inj. of CEV samples*	%CV of peak area in reference sample** injected between real samples (n=4)	%CV of peak symmetry in CEV samples* (n=30)	%CV of peak width in CEV samples* (n=30)	%CV of peak retention time in CEV samples* (n=30)	%CV of peak area in CEV samples* (n=30)	%CV of peak area in CEV samples* discarding first 5 injections (n=25)
Lipid standards	C15 Ceramide-d7	2.3	7%	18%	11%	0.78%	12%	9%
	18:1 (d7) CE	3	12%	23%	12%	0.26%	15%	12%
	d18:1-18:1 (d9) SM	2.5	10%	14%	8%	0.87%	11%	7%
	15:0-18:1 (d7) PC	2.4	9%	16%	19%	0.82%	13%	8%
	15:0-18:1 (d7)-15:0 TG	2.8	6%	17%	10%	0.25%	15%	9%
CEV lipids	MGDG 43:3	-	-	19%	8%	0.82%	22%	10%
	SQDG 32:0	-	-	14%	11%	1.04%	18%	9%
	DGDG 34:1	-	-	27%	9%	0.80%	14%	8%

*CEV samples spiked with lipid standards
** reference sample containing lipid standards in methanol

Table S7. Lipid profiles of FBSEV obtained by LC-MS analysis of LLE extracts (3 extraction replicates and 3 LC-MS replicates, 20 µL of EV sample for extraction, chromatographic condition 2).

				% relative amount										
Name	Formula	Mass (avg)	RT (avg)	LLE A _1	LLE A_2	LLE A_3	LLE B_1	LLE B _2	LLE B_3	LLE C_1	LLE C_2	LLE C_3	average relative amount	%CV of peak area
CE 14:0	C41 H72 O2	596.55 14	16.8	0.76%	0.75%	0.74%	0.72%	0.70%	0.70%	0.75%	0.68%	0.73%	0.73%	9.5%
CE 16:0	C43 H76 O2	624.58 24	18.1	1.74%	1.44%	1.16%	1.18%	1.13%	1.11%	0.91%	1.09%	1.11%	1.21%	16.1%
CE 16:1	C43 H74 O2	622.56 73	17.0	6.49%	6.41%	6.34%	6.20%	6.09%	6.09%	6.59%	6.12%	6.24%	6.29%	10.5%
CE 18:1	C45 H78 O2	650.59 87	18.4	23.62 %	23.56 %	23.62 %	23.21 %	22.85 %	22.69 %	17.56%	23.03 %	21.76 %	22.43%	15.2%
CE 18:2	C45 H76 O2	648.58 31	17.4	21.42 %	21.74 %	21.95 %	21.95 %	22.41 %	21.98 %	23.75%	22.01 %	20.86 %	22.01%	14.0%
CE 18:3	C45 H74 O2	646.56 72	16.6	3.41%	3.53%	3.39%	3.50%	3.66%	3.65%	3.96%	3.64%	3.88%	3.63%	13.3%
CE 20:3	C47 H78 O2	674.59 78	17.8	3.79%	3.67%	3.61%	4.04%	3.91%	4.02%	3.88%	4.09%	4.30%	3.92%	14.0%
CE 20:4	C47 H76 O2	672.58 56	17.2	24.88 %	24.60 %	24.85 %	24.75 %	24.70 %	24.93 %	26.67%	24.82 %	25.43 %	25.07%	11.9%
CE 20:5	C47 H74 O2	670.56 74	16.4	6.75%	6.88%	6.91%	6.91%	6.91%	7.06%	7.66%	6.95%	7.14%	7.02%	12.6%
CE 22:6	C49 H76 O2	696.58 28	16.9	7.00%	7.28%	7.30%	7.41%	7.50%	7.64%	8.14%	7.44%	8.37%	7.56%	12.6%
cholesterol	C27 H46 O	386.35 38	5.7	0.15%	0.13%	0.14%	0.13%	0.13%	0.13%	0.14%	0.13%	0.18%	0.14%	6.7%
total peak area CE				1.91E +08	2.13E +08	2.15E +08	2.44E +08	2.63E +08	2.51E +08	223931 700	2.52E +08	1.86E +08		12.2%
LPC 20:1	C28 H56 N O7 P	549.37 82	1.701 79	0.15%	0.14%	0.14%	0.15%	0.14%	0.15%	0.14%	0.15%	0.15%	0.14%	8.15%
LPC 20:4	C28 H50 N O7 P	543.33 2	0.988 57	1.48%	1.44%	1.51%	1.45%	1.51%	1.48%	1.49%	1.50%	1.53%	1.49%	7.05%

LPC 20:5	C28 H48 N O7 P	541.3159	0.85809	0.24%	0.24%	0.24%	0.24%	0.25%	0.24%	0.24%	0.24%	0.24%	0.24%	7.59%
LPC 14:0	C22 H46 N O7 P	467.3008	0.82314	0.17%	0.16%	0.17%	0.17%	0.17%	0.17%	0.17%	0.17%	0.18%	0.17%	7.79%
LPC 16:0	C24 H50 N O7 P	495.3321	1.10322	4.56%	4.44%	4.61%	4.71%	4.64%	4.49%	4.59%	4.74%	4.78%	4.62%	7.76%
LPC 16:1	C24 H48 N O7 P	493.3165	0.90202	0.74%	0.72%	0.74%	0.73%	0.75%	0.74%	0.75%	0.75%	0.77%	0.74%	7.08%
LPC 18:0	C26 H54 N O7 P	523.3633	1.57054	4.12%	3.97%	4.09%	4.01%	4.17%	3.98%	4.16%	4.10%	4.28%	4.10%	6.86%
LPC 18:1	C26 H52 N O7 P	521.348	1.20178	4.10%	4.05%	4.14%	4.22%	4.19%	4.09%	4.09%	4.10%	4.10%	4.12%	7.86%
LPC 18:2	C26 H50 N O7 P	519.3314	1.00416	0.55%	0.54%	0.55%	0.54%	0.56%	0.54%	0.55%	0.54%	0.56%	0.55%	7.09%
LPC 20:3	C28 H52 N O7 P	545.347	1.12851	0.54%	0.55%	0.55%	0.55%	0.57%	0.56%	0.54%	0.56%	0.57%	0.55%	8.08%
LPC22:4	C30 H54 N O7 P	571.3625	1.29027	0.10%	0.09%	0.10%	0.10%	0.09%	0.09%	0.10%	0.10%	0.09%	0.10%	6.82%
LPC22:5	C30 H52 N O7 P	569.3474	1.08651	1.00%	0.99%	1.04%	1.02%	1.04%	1.01%	1.03%	1.02%	1.04%	1.02%	7.45%
LPC22:6	C30 H50 N O7 P	567.3319	0.98928	1.04%	1.04%	1.05%	1.02%	1.07%	1.05%	1.06%	1.07%	1.07%	1.05%	7.03%
PC 30:0	C38 H76 N O8 P	705.5289	6.64491	0.36%	0.41%	0.37%	0.41%	0.41%	0.41%	0.37%	0.41%	0.37%	0.39%	12.34%
PC 31:0	C39 H78 N O8 P	719.5446	7.36955	0.23%	0.21%	0.22%	0.21%	0.21%	0.23%	0.21%	0.23%	0.23%	0.22%	7.50%
PC 32:0	C40 H80 N O8 P	733.5607	8.07957	3.46%	3.47%	3.45%	3.45%	3.47%	3.46%	3.36%	3.46%	3.51%	3.45%	7.06%
PC 32:1	C40 H78 N O8 P	731.5447	7.096	1.49%	1.37%	1.48%	1.40%	1.38%	1.40%	1.48%	1.40%	1.47%	1.43%	5.74%
PC 33:1	C41 H80 N O8 P	745.56	7.76073	0.83%	0.84%	0.85%	0.81%	0.80%	0.83%	0.80%	0.78%	0.85%	0.82%	6.21%
PC 34:0	C42 H84 N O8 P	761.5912	9.47107	1.36%	1.35%	1.38%	1.37%	1.34%	1.34%	1.35%	1.36%	1.41%	1.36%	6.55%

PC 34:1	C42 H82 N O8 P	759.5771	8.43876	18.51%	18.63%	18.65%	18.36%	18.56%	18.41%	18.77%	18.49%	18.86%	18.58%	6.52%
PC 34:2	C42 H80 N O8 P	757.5607	7.5555	2.26%	2.27%	2.27%	2.28%	2.30%	2.28%	2.29%	2.26%	2.28%	2.28%	7.35%
PC 34:3	C42 H78 N O8 P	755.542	8.08323	0.40%	0.41%	0.41%	0.39%	0.41%	0.39%	0.40%	0.40%	0.40%	0.40%	6.17%
PC 34:4	C42 H76 N O8 P	753.527	7.08716	0.19%	0.17%	0.17%	0.16%	0.18%	0.16%	0.17%	0.16%	0.17%	0.17%	7.47%
PC 35:1	C43 H84 N O8 P	773.5912	9.14384	1.42%	1.44%	1.42%	1.39%	1.35%	1.41%	1.43%	1.40%	1.42%	1.41%	5.75%
PC 35:2	C43 H82 N O8 P	771.5758	8.17453	0.38%	0.40%	0.38%	0.39%	0.40%	0.39%	0.35%	0.39%	0.39%	0.38%	8.76%
PC 36:1	C44 H86 N O8 P	787.608	9.81671	13.41%	13.51%	13.50%	13.30%	13.25%	13.28%	13.54%	13.45%	13.48%	13.41%	6.28%
PC 36:2	C44 H84 N O8 P	785.592	8.81034	5.71%	5.75%	5.72%	5.72%	5.76%	5.69%	5.80%	5.74%	5.81%	5.74%	6.86%
PC 36:4	C44 H80 N O8 P	781.5607	7.57575	2.20%	2.20%	2.23%	2.24%	2.25%	2.25%	2.24%	2.21%	2.22%	2.22%	7.73%
PC 38:1	C46 H90 N O8 P	815.638	11.0728	0.32%	0.29%	0.24%	0.32%	0.35%	0.29%	0.32%	0.28%	0.29%	0.30%	15.47%
PC 38:3	C46 H86 N O8 P	811.6073	9.50433	4.42%	4.39%	3.91%	4.37%	3.89%	4.41%	3.96%	4.33%	4.36%	4.23%	8.99%
PC 38:4	C46 H84 N O8 P	809.5921	8.98569	5.76%	5.89%	5.88%	5.84%	5.77%	5.73%	5.42%	5.87%	4.52%	5.63%	10.88%
PC 38:5	C46 H82 N O8 P	807.5762	7.93022	4.70%	4.60%	4.74%	4.91%	4.55%	4.77%	4.78%	4.53%	4.59%	4.69%	8.23%
PC 38:6	C46 H80 N O8 P	805.5608	7.52036	2.24%	2.38%	2.29%	2.51%	2.60%	2.55%	2.48%	2.40%	2.55%	2.44%	11.45%
PC 38:7	C46 H78 N O8 P	803.5417	7.56777	0.25%	0.27%	0.25%	0.25%	0.26%	0.25%	0.25%	0.26%	0.26%	0.25%	7.14%
PC 40:4	C48 H88 N O8 P	837.6214	10.0234	0.41%	0.38%	0.35%	0.39%	0.41%	0.37%	0.36%	0.37%	0.30%	0.37%	12.68%
PC 40:5	C48 H86 N O8 P	835.6073	9.32433	4.60%	4.64%	4.61%	4.34%	4.65%	4.78%	4.82%	4.79%	4.63%	4.65%	7.08%

PC 40:6	C48 H84 N O8 P	833.5922	8.9232	4.06%	3.99%	3.91%	4.00%	4.02%	3.95%	3.91%	3.74%	3.94%	3.95%	7.57%
PC 40:7	C48 H82 N O8 P	831.5746	7.87575	0.51%	0.49%	0.51%	0.52%	0.50%	0.51%	0.51%	0.50%	0.50%	0.51%	7.59%
PC 40:8	C48 H80 N O8 P	829.5575	7.93709	0.47%	0.60%	0.54%	0.51%	0.55%	0.55%	0.52%	0.55%	0.54%	0.54%	9.54%
PC 40:9	C48 H78 N O8 P	827.5421	7.52102	0.26%	0.28%	0.29%	0.29%	0.27%	0.26%	0.26%	0.26%	0.29%	0.27%	8.09%
PC 42:8	C50 H84 N O8 P	857.5888	9.3197	0.56%	0.52%	0.58%	0.51%	0.51%	0.57%	0.49%	0.51%	0.57%	0.53%	8.12%
PC 42:9	C50 H82 N O8 P	855.5731	8.92189	0.45%	0.47%	0.47%	0.46%	0.46%	0.46%	0.46%	0.46%	0.46%	0.46%	6.81%
total peak area PC				4.74E+08	4.81E+08	4.77E+08	5.63E+08	5.53E+08	5.58E+08	506210791	5.1E+08	5.03E+08		6.97%
PC O-34:1	C42 H84 N O7 P	745.5954	9.29252	37.40%	38.19%	37.20%	37.96%	37.65%	37.25%	37.66%	37.73%	37.19%	37.58%	7.58%
PC O-34:2	C42 H82 N O7 P	743.5805	9.1368	21.77%	21.86%	21.54%	23.48%	21.87%	21.50%	21.52%	22.88%	21.66%	22.01%	8.91%
PC O-36:5	C44 H80 N O7 P	765.5654	8.25182	7.12%	6.21%	6.05%	7.09%	7.66%	6.65%	7.36%	6.60%	7.23%	6.88%	12.80%
PC O-38:6	C46 H82 N O7 P	791.5806	8.59212	15.44%	15.96%	15.62%	13.23%	15.37%	15.23%	15.77%	15.12%	15.55%	15.26%	6.66%
PC O-38:7	C46 H80 N O7 P	789.5655	8.18019	10.69%	10.32%	12.23%	10.73%	9.82%	12.13%	9.86%	10.26%	10.28%	10.70%	11.79%
LPC O-16:0	C24 H52 N O6 P	481.3518	1.34423	2.50%	2.55%	2.51%	2.64%	2.56%	2.47%	2.65%	2.60%	2.75%	2.58%	7.96%
LPC O-16:1	C24 H50 N O6 P	479.3375	1.27815	3.81%	3.67%	3.53%	3.61%	3.86%	3.74%	3.87%	3.49%	3.97%	3.73%	8.98%
LPC O-18:0	C26 H56 N O6 P	509.3831	1.94714	1.29%	1.25%	1.32%	1.25%	1.21%	1.03%	1.31%	1.32%	1.39%	1.26%	6.36%
total peak area PC-O				11616697	11875582	11861557	13725263	13905702	14091686	12565418	12511230	12281486		7.47%
SM 32:1; O2	C37 H75 N2 O6 P	674.5343	4.93802	1.10%	1.07%	1.09%	1.14%	1.14%	1.11%	1.10%	1.09%	1.03%	1.10%	9.07%

SM 33:1; O2	C38 H77 N2 O6 P	688.5499	5.64214	1.67%	1.64%	1.65%	1.73%	1.72%	1.71%	1.75%	1.69%	1.71%	1.69%	8.56%
SM 34:0; O2	C39 H81 N2 O6 P	704.5812	6.91818	2.12%	2.05%	2.06%	2.06%	2.07%	2.10%	2.08%	2.04%	2.04%	2.07%	7.02%
SM 34:1; O2	C39 H79 N2 O6 P	702.5668	6.3374	37.15%	36.99%	37.20%	37.21%	37.47%	37.16%	37.47%	37.12%	37.48%	37.25%	7.02%
SM 34:2; O2	C39 H77 N2 O6 P	700.5503	5.38142	3.80%	3.78%	3.80%	3.88%	3.93%	3.79%	3.83%	3.80%	3.81%	3.82%	7.72%
SM 35:1; O2	C40 H81 N2 O6 P	716.581	7.08216	1.10%	1.06%	1.08%	1.10%	1.08%	1.09%	1.11%	1.05%	1.06%	1.08%	7.55%
SM 36:1; O2	C41 H83 N2 O6 P	730.5974	7.8	7.15%	7.18%	7.08%	7.12%	7.19%	7.20%	7.17%	7.18%	7.12%	7.15%	7.07%
SM 36:2; O2	C41 H81 N2 O6 P	728.5813	6.83591	2.51%	2.46%	2.48%	2.51%	2.48%	2.45%	2.45%	2.47%	2.45%	2.47%	6.98%
SM 38:1; O2	C43 H87 N2 O6 P	758.6271	9.25233	2.39%	2.25%	2.35%	2.26%	2.45%	2.28%	2.31%	2.41%	2.24%	2.33%	7.59%
SM 39:1; O2	C44 H89 N2 O6 P	772.6419	9.93879	1.01%	1.06%	0.95%	0.95%	1.00%	0.96%	0.98%	0.96%	1.06%	0.99%	6.28%
SM 40:1; O2	C45 H91 N2 O6 P	786.6594	10.5664	11.40%	11.49%	11.46%	11.54%	11.39%	11.35%	11.44%	11.42%	11.33%	11.43%	6.87%
SM 41:1; O2	C46 H93 N2 O6 P	800.6741	11.2079	2.35%	2.36%	2.30%	2.33%	2.32%	2.34%	2.31%	2.30%	2.33%	2.33%	6.82%
SM 42:1; O2	C47 H95 N2 O6 P	814.6905	11.8038	7.07%	7.19%	7.17%	6.66%	6.84%	7.07%	6.62%	7.01%	7.03%	6.96%	5.74%
SM 42:2; O2	C47 H93 N2 O6 P	812.6755	10.742	19.19%	19.42%	19.32%	19.51%	18.91%	19.38%	19.39%	19.46%	19.33%	19.32%	6.90%
total peak area SM				1.02E+08	1.04E+08	1.04E+08	1.22E+08	1.19E+08	1.2E+08	110360108	1.11E+08	1.1E+08		6.86%

Table S8. Lipid profiles of FBSEV obtained by LC-MS analysis of single-phase extracts (3 extraction replicates and 3 LC-MS replicates, 10 μL of EV sample for extraction, chromatographic condition 2).

				% relative amount										
Name	Formula	Mass (avg)	RT (avg)	SIN_PH A_1	SIN_PH A_2	SIN_PH A_3	SIN_PH B_1	SIN_PH B_2	SIN_PH B_3	SIN_PH C_1	SIN_PH C_2	SIN_PH C_3	average relative amount	%CV of peak area
CE 14:0	C41 H72 O2	596.5514	16.76	0.9%	0.9%	0.9%	0.7%	0.7%	0.6%	0.8%	0.8%	0.8%	0.8%	13.8%
CE 16:0	C43 H76 O2	624.5824	18.12	0.8%	0.9%	0.8%	0.6%	0.5%	0.6%	0.5%	0.6%	0.4%	0.6%	13.8%
CE 16:1	C43 H74 O2	622.5673	17.02	7.9%	8.0%	7.9%	6.8%	6.7%	6.3%	7.3%	7.4%	7.5%	7.3%	15.2%
CE 18:1	C45 H78 O2	650.5987	18.44	23.2%	23.6%	23.6%	20.1%	20.1%	24.1%	22.9%	23.2%	23.6%	22.7%	15.1%
CE 18:2	C45 H76 O2	648.5831	17.41	20.7%	20.4%	20.5%	20.4%	20.4%	19.0%	20.7%	20.9%	20.9%	20.4%	20.4%
CE 18:3	C45 H74 O2	646.5672	16.64	3.2%	3.1%	3.1%	3.4%	3.4%	3.2%	3.2%	3.2%	3.2%	3.2%	23.7%
CE 20:3	C47 H78 O2	674.5978	17.84	3.3%	3.4%	3.4%	3.3%	3.5%	4.1%	3.7%	3.6%	3.5%	3.5%	21.1%
CE 20:4	C47 H76 O2	672.5856	17.20	26.3%	25.7%	25.8%	28.6%	28.6%	26.3%	26.5%	26.2%	26.0%	26.7%	24.4%
CE 20:5	C47 H74 O2	670.5674	16.41	6.6%	6.8%	6.8%	8.0%	8.0%	7.7%	7.0%	6.9%	6.9%	7.2%	27.3%
CE 22:6	C49 H76 O2	696.5828	16.87	6.7%	6.8%	6.8%	7.9%	8.0%	7.6%	7.1%	7.0%	6.9%	7.2%	26.7%
cholesterol	C27 H46 O	386.3538	5.74	0.3%	0.4%	0.3%	0.3%	0.3%	0.4%	0.3%	0.3%	0.3%	0.3%	21.7%
total peak area CE				61547824	57427306	57911739	96599010	99918731	75928648	83150847	77641544	77862902		20.3%
LPC 20:1	C28 H56 N O7 P	549.3782	1.701791	0.22%	0.22%	0.21%	0.19%	0.19%	0.19%	0.20%	0.21%	0.21%	0.20%	10.1%

LPC 20:4	C28 H50 N O7 P	543.332	0.988565	2.57%	2.21%	2.42%	2.00%	2.01%	2.03%	2.30%	2.27%	2.29%	2.23%	9.7%
LPC 20:5	C28 H48 N O7 P	541.3159	0.858093	0.45%	0.30%	0.39%	0.33%	0.33%	0.34%	0.37%	0.37%	0.38%	0.36%	14.3%
LPC 14:0	C22 H46 N O7 P	467.3008	0.82314	0.36%	0.26%	0.33%	0.27%	0.27%	0.28%	0.31%	0.31%	0.31%	0.30%	13.1%
LPC 16:0	C24 H50 N O7 P	495.3321	1.103217	8.19%	7.36%	7.68%	6.62%	6.58%	6.59%	7.39%	7.32%	7.45%	7.24%	10.0%
LPC 16:1	C24 H48 N O7 P	493.3165	0.902023	1.46%	1.09%	1.34%	1.10%	1.10%	1.12%	1.27%	1.26%	1.27%	1.22%	11.6%
LPC 18:0	C26 H54 N O7 P	523.3633	1.570543	6.46%	6.63%	6.46%	5.53%	5.34%	5.46%	6.01%	6.13%	6.13%	6.02%	9.3%
LPC 18:1	C26 H52 N O7 P	521.348	1.201783	6.97%	6.45%	6.58%	5.56%	5.40%	5.66%	6.34%	6.16%	6.19%	6.15%	9.5%
LPC 18:2	C26 H50 N O7 P	519.3314	1.004159	0.92%	0.78%	0.85%	0.71%	0.72%	0.73%	0.82%	0.82%	0.81%	0.80%	10.1%
LPC 20:3	C28 H52 N O7 P	545.347	1.128512	0.89%	0.85%	0.82%	0.69%	0.74%	0.74%	0.82%	0.83%	0.80%	0.80%	9.6%
LPC22:4	C30 H54 N O7 P	571.3625	1.290273	0.16%	0.15%	0.15%	0.12%	0.12%	0.13%	0.14%	0.15%	0.14%	0.14%	7.5%
LPC22:5	C30 H52 N O7 P	569.3474	1.086511	1.67%	1.45%	1.57%	1.31%	1.32%	1.34%	1.50%	1.51%	1.51%	1.46%	9.6%
LPC22:6	C30 H50 N O7 P	567.3319	0.989279	1.74%	1.42%	1.64%	1.33%	1.36%	1.36%	1.53%	1.51%	1.54%	1.49%	10.5%
PC 30:0	C38 H76 N O8 P	705.5289	6.644907	0.31%	0.34%	0.35%	0.34%	0.36%	0.38%	0.38%	0.37%	0.37%	0.35%	17.8%
PC 31:0	C39 H78 N O8 P	719.5446	7.369545	0.16%	0.17%	0.19%	0.21%	0.21%	0.19%	0.20%	0.20%	0.21%	0.19%	22.2%
PC 32:0	C40 H80 N O8 P	733.5607	8.079574	2.70%	2.77%	2.73%	2.99%	3.05%	3.08%	2.91%	2.85%	2.87%	2.88%	20.6%
PC 32:1	C40 H78 N O8 P	731.5447	7.096	1.41%	1.36%	1.33%	1.35%	1.41%	1.45%	1.43%	1.42%	1.35%	1.39%	17.3%
PC 33:1	C41 H80 N O8 P	745.56	7.760733	0.74%	0.81%	0.75%	0.79%	0.81%	0.76%	0.75%	0.78%	0.79%	0.78%	17.8%

PC 34:0	C42 H84 N O8 P	761.5912	9.471067	0.92%	0.96%	0.96%	1.11%	1.10%	1.14%	1.03%	0.98%	1.00%	1.02%	23.9%
PC 34:1	C42 H82 N O8 P	759.5771	8.438756	16.27%	16.88%	16.56%	16.94%	17.28%	17.39%	17.19%	16.98%	17.15%	16.96%	17.4%
PC 34:2	C42 H80 N O8 P	757.5607	7.5555	2.10%	2.19%	2.15%	2.21%	2.21%	2.22%	2.20%	2.13%	2.16%	2.17%	17.7%
PC 34:3	C42 H78 N O8 P	755.542	8.083227	0.32%	0.32%	0.33%	0.32%	0.35%	0.35%	0.32%	0.33%	0.33%	0.33%	18.7%
PC 34:4	C42 H76 N O8 P	753.527	7.087163	0.15%	0.18%	0.15%	0.16%	0.15%	0.17%	0.16%	0.14%	0.17%	0.16%	18.1%
PC 35:1	C43 H84 N O8 P	773.5912	9.143837	1.11%	1.18%	1.17%	1.25%	1.24%	1.26%	1.23%	1.21%	1.21%	1.21%	19.1%
PC 35:2	C43 H82 N O8 P	771.5758	8.174535	0.33%	0.35%	0.36%	0.37%	0.38%	0.38%	0.38%	0.40%	0.36%	0.37%	18.3%
PC 36:1	C44 H86 N O8 P	787.608	9.816711	9.95%	10.37%	10.24%	11.43%	11.61%	11.72%	11.08%	10.86%	10.95%	10.91%	21.3%
PC 36:2	C44 H84 N O8 P	785.592	8.810341	4.72%	4.88%	4.81%	5.13%	5.18%	5.19%	5.02%	4.98%	4.93%	4.98%	19.2%
PC 36:4	C44 H80 N O8 P	781.5607	7.57575	2.12%	2.15%	2.16%	2.24%	2.17%	2.21%	2.20%	2.17%	2.17%	2.18%	17.4%
PC 38:1	C46 H90 N O8 P	815.638	11.07277	0.20%	0.19%	0.17%	0.26%	0.24%	0.23%	0.21%	0.24%	0.20%	0.21%	27.9%
PC 38:3	C46 H86 N O8 P	811.6073	9.504333	3.33%	3.51%	3.46%	3.84%	3.87%	3.79%	3.30%	3.26%	3.57%	3.55%	22.9%
PC 38:4	C46 H84 N O8 P	809.5921	8.985689	4.43%	5.02%	4.97%	5.34%	5.40%	4.77%	4.55%	5.16%	3.80%	4.83%	23.6%
PC 38:5	C46 H82 N O8 P	807.5762	7.930222	4.52%	4.91%	4.55%	4.85%	4.91%	4.96%	4.40%	4.61%	4.58%	4.70%	19.9%
PC 38:6	C46 H80 N O8 P	805.5608	7.520356	2.37%	2.43%	2.39%	2.47%	2.37%	2.06%	2.19%	2.18%	2.47%	2.33%	17.0%
PC 38:7	C46 H78 N O8 P	803.5417	7.567773	0.24%	0.23%	0.24%	0.23%	0.22%	0.25%	0.24%	0.24%	0.24%	0.24%	15.3%
PC 40:4	C48 H88 N O8 P	837.6214	10.02341	0.31%	0.31%	0.27%	0.31%	0.33%	0.33%	0.31%	0.20%	0.30%	0.30%	25.7%

PC 40:5	C48 H86 N O8 P	835.6073	9.324326	3.87%	3.84%	3.96%	4.21%	3.95%	3.92%	3.71%	3.96%	4.15%	3.95%	18.3%
PC 40:6	C48 H84 N O8 P	833.5922	8.9232	3.26%	3.31%	3.28%	3.75%	3.54%	3.70%	3.44%	3.37%	3.51%	3.46%	21.2%
PC 40:7	C48 H82 N O8 P	831.5746	7.87575	0.49%	0.52%	0.50%	0.50%	0.51%	0.50%	0.49%	0.48%	0.48%	0.50%	17.8%
PC 40:8	C48 H80 N O8 P	829.5575	7.937091	0.53%	0.53%	0.46%	0.52%	0.49%	0.50%	0.53%	0.49%	0.49%	0.51%	16.9%
PC 40:9	C48 H78 N O8 P	827.5421	7.521023	0.24%	0.25%	0.27%	0.25%	0.25%	0.25%	0.26%	0.25%	0.25%	0.25%	15.3%
PC 42:8	C50 H84 N O8 P	857.5888	9.319705	0.46%	0.48%	0.42%	0.49%	0.49%	0.50%	0.44%	0.49%	0.49%	0.47%	19.8%
PC 42:9	C50 H82 N O8 P	855.5731	8.921886	0.39%	0.40%	0.40%	0.39%	0.43%	0.43%	0.43%	0.41%	0.41%	0.41%	17.9%
total peak area PC				351955304	338137651	3.38E+08	4.72E+08	4.59E+08	4.59E+08	3.39E+08	3.36E+08	334709699		16.3%
PC O-34:1	C42 H84 N O7 P	745.5954	9.292523	31.7%	31.8%	31.9%	33.4%	33.8%	32.8%	33.8%	33.6%	30.7%	32.6%	18.9%
PC O-34:2	C42 H82 N O7 P	743.5805	9.136795	19.8%	20.4%	20.7%	21.0%	23.3%	21.4%	21.5%	21.5%	21.1%	21.2%	19.6%
PC O-36:5	C44 H80 N O7 P	765.5654	8.251818	6.4%	6.5%	6.9%	6.9%	6.0%	7.4%	7.9%	7.7%	7.7%	7.0%	17.5%
PC O-38:6	C46 H82 N O7 P	791.5806	8.592116	15.4%	15.7%	15.5%	15.4%	13.2%	15.2%	13.3%	15.9%	15.5%	15.0%	17.2%
PC O-38:7	C46 H80 N O7 P	789.5655	8.180186	12.8%	12.7%	12.3%	12.3%	12.3%	12.2%	11.0%	9.1%	12.5%	11.9%	21.5%
LPC O-16:0	C24 H52 N O6 P	481.3518	1.344233	4.3%	4.2%	4.2%	3.4%	3.6%	3.5%	4.0%	3.8%	4.0%	3.9%	9.4%
LPC O-16:1	C24 H50 N O6 P	479.3375	1.278152	7.7%	6.8%	6.8%	5.8%	6.2%	5.8%	6.7%	6.7%	6.8%	6.6%	10.3%
LPC O-18:0	C26 H56 N O6 P	509.3831	1.947136	1.9%	1.9%	1.7%	1.6%	1.5%	1.6%	1.9%	1.8%	1.8%	1.7%	11.6%
total peak area PC-O				8696691	8479057	8484289	12011440	11088533	11948526	8478383	8310145	8383272		17.1%

SM 32:1; O2	C37 H75 N2 O6 P	674.5343	4.938023	1.5%	1.5%	1.5%	1.3%	1.2%	1.3%	1.4%	1.4%	1.4%	1.4%	14.9%
SM 33:1; O2	C38 H77 N2 O6 P	688.5499	5.64214	2.1%	2.0%	2.1%	1.9%	1.8%	1.9%	1.9%	2.0%	2.0%	2.0%	17.7%
SM 34:0; O2	C39 H81 N2 O6 P	704.5812	6.918182	2.2%	2.3%	2.2%	2.1%	2.1%	2.2%	2.2%	2.2%	2.2%	2.2%	19.0%
SM 34:1; O2	C39 H79 N2 O6 P	702.5668	6.3374	43.6%	43.3%	43.3%	39.7%	40.2%	40.5%	42.0%	42.0%	42.0%	41.8%	17.7%
SM 34:2; O2	C39 H77 N2 O6 P	700.5503	5.381422	5.2%	5.3%	5.2%	4.6%	4.5%	4.5%	4.9%	4.9%	4.9%	4.9%	15.2%
SM 35:1; O2	C40 H81 N2 O6 P	716.581	7.082163	1.1%	1.2%	1.1%	1.1%	1.1%	1.1%	1.2%	1.1%	1.1%	1.1%	20.1%
SM 36:1; O2	C41 H83 N2 O6 P	730.5974	7.8	7.2%	7.1%	7.1%	7.5%	7.1%	7.2%	7.3%	7.2%	7.2%	7.2%	22.1%
SM 36:2; O2	C41 H81 N2 O6 P	728.5813	6.835907	3.0%	3.0%	3.0%	2.7%	2.7%	2.8%	3.0%	3.0%	2.9%	2.9%	16.9%
SM 38:1; O2	C43 H87 N2 O6 P	758.6271	9.252326	2.1%	2.5%	2.1%	2.2%	2.3%	2.1%	2.1%	2.3%	2.1%	2.2%	22.0%
SM 39:1; O2	C44 H89 N2 O6 P	772.6419	9.938791	0.8%	0.8%	0.8%	0.9%	0.7%	0.9%	1.0%	0.9%	0.8%	0.9%	22.7%
SM 40:1; O2	C45 H91 N2 O6 P	786.6594	10.5664	8.4%	8.7%	8.8%	10.0%	10.1%	10.0%	9.3%	9.2%	9.7%	9.4%	27.2%
SM 41:1; O2	C46 H93 N2 O6 P	800.6741	11.20788	1.7%	1.8%	1.8%	2.0%	2.0%	2.0%	1.9%	1.9%	1.9%	1.9%	27.3%
SM 42:1; O2	C47 H95 N2 O6 P	814.6905	11.80381	5.0%	4.9%	4.8%	6.0%	6.1%	5.6%	5.3%	5.4%	5.2%	5.4%	29.7%
SM 42:2; O2	C47 H93 N2 O6 P	812.6755	10.74195	16.1%	15.8%	16.1%	17.9%	17.8%	17.9%	16.6%	16.7%	16.6%	16.8%	26.2%
total peak area SM				59357095	59386994	58115512	90566549	88439444	88829040	62121867	60211216	60659938		21.1%

Table S9. Lipid profiles of FBSEV obtained by LC-MS analysis of SPE (3 extraction replicates and 3 LC-MS replicates, 50 μL of EV sample for extraction, chromatographic condition 2).

				% relative amount										
Name	Formula	Mass (avg)	RT (avg)	SPE A_1	SPE A_2	SPE A_3	SPE B_1	SPE B_2	SPE B_3	SPE C_1	SPE C_2	SPE C_3	average relative amount	%CV of peak area
LPC 20:1	C28 H56 N O7 P	549.3782	1.701791	0.3%	0.3%	0.3%	0.3%	0.3%	0.3%	0.3%	0.3%	0.3%	0.3%	8.6%
LPC 20:4	C28 H50 N O7 P	543.332	0.988565	3.1%	3.2%	3.2%	3.3%	3.3%	3.3%	3.2%	3.2%	3.2%	3.2%	7.3%
LPC 20:5	C28 H48 N O7 P	541.3159	0.858093	0.4%	0.4%	0.4%	0.4%	0.4%	0.4%	0.4%	0.4%	0.4%	0.4%	9.2%
LPC 14:0	C22 H46 N O7 P	467.3008	0.82314	0.3%	0.3%	0.3%	0.3%	0.3%	0.3%	0.3%	0.3%	0.3%	0.3%	8.3%
LPC 16:0	C24 H50 N O7 P	495.3321	1.103217	6.8%	6.7%	6.5%	6.9%	7.3%	7.4%	7.5%	7.4%	7.4%	7.1%	4.2%
LPC 16:1	C24 H48 N O7 P	493.3165	0.902023	1.1%	1.1%	1.1%	1.1%	1.1%	1.1%	1.1%	1.1%	1.1%	1.1%	8.1%
LPC 18:0	C26 H54	523.3633	1.570543	8.3%	8.5%	8.6%	8.7%	9.2%	9.0%	8.9%	8.6%	8.8%	8.7%	6.4%

	N O7 P													
LPC 18:1	C26 H52 N O7 P	521.348	1.201783	10.3%	10.2%	10.0%	10.8%	11.0%	11.2%	10.8%	10.9%	10.9%	10.7%	4.7%
LPC 18:2	C26 H50 N O7 P	519.3314	1.004159	1.3%	1.3%	1.3%	1.3%	1.3%	1.3%	1.3%	1.3%	1.3%	1.3%	6.5%
LPC 20:3	C28 H52 N O7 P	545.347	1.128512	1.6%	1.6%	1.5%	1.6%	1.7%	1.7%	1.5%	1.6%	1.6%	1.6%	6.8%
LPC22:4	C30 H54 N O7 P	571.3625	1.290273	0.3%	0.3%	0.3%	0.3%	0.3%	0.3%	0.2%	0.3%	0.2%	0.3%	8.3%
LPC22:5	C30 H52 N O7 P	569.3474	1.086511	2.2%	2.2%	2.1%	2.3%	2.3%	2.3%	2.2%	2.3%	2.3%	2.2%	5.2%
LPC22:6	C30 H50 N O7 P	567.3319	0.989279	1.8%	1.8%	1.8%	1.8%	1.8%	1.9%	1.8%	1.8%	1.8%	1.8%	7.7%
PC 30:0	C38 H76 N O8 P	705.5289	6.644907	0.4%	0.3%	0.4%	0.3%	0.3%	0.3%	0.3%	0.3%	0.3%	0.3%	14.4%
PC 31:0	C39 H78 N O8 P	719.5446	7.369545	0.2%	0.2%	0.2%	0.2%	0.2%	0.1%	0.2%	0.2%	0.2%	0.2%	16.0%

PC 32:0	C40 H80 N O8 P	733.5607	8.079574	2.8%	2.8%	2.8%	2.7%	2.6%	2.7%	2.6%	2.7%	2.6%	2.7%	11.1%
PC 32:1	C40 H78 N O8 P	731.5447	7.096	1.2%	1.2%	1.2%	1.2%	1.1%	1.2%	1.1%	1.2%	1.1%	1.2%	10.5%
PC 33:1	C41 H80 N O8 P	745.56	7.760733	0.6%	0.7%	0.7%	0.7%	0.6%	0.6%	0.6%	0.7%	0.6%	0.6%	10.0%
PC 34:0	C42 H84 N O8 P	761.5912	9.471067	1.1%	1.1%	1.1%	1.0%	1.0%	1.0%	1.0%	1.0%	1.0%	1.0%	12.9%
PC 34:1	C42 H82 N O8 P	759.5771	8.438756	14.9%	14.7%	14.9%	14.4%	14.2%	14.3%	14.3%	14.4%	14.2%	14.5%	10.0%
PC 34:2	C42 H80 N O8 P	757.5607	7.5555	1.9%	1.9%	1.9%	1.8%	1.8%	1.8%	1.8%	1.8%	1.8%	1.8%	10.7%
PC 34:3	C42 H78 N O8 P	755.542	8.083227	0.3%	0.3%	0.3%	0.3%	0.3%	0.3%	0.3%	0.3%	0.3%	0.3%	10.8%
PC 34:4	C42 H76 N O8 P	753.527	7.087163	0.1%	0.1%	0.1%	0.1%	0.1%	0.1%	0.1%	0.1%	0.1%	0.1%	11.1%

PC 35:1	C43 H84 N O8 P	773.5912	9.143837	1.0%	1.1%	1.1%	1.1%	1.0%	1.0%	1.0%	1.1%	1.0%	1.1%	10.0%
PC 35:2	C43 H82 N O8 P	771.5758	8.174535	0.3%	0.3%	0.3%	0.3%	0.3%	0.3%	0.3%	0.3%	0.3%	0.3%	11.4%
PC 36:1	C44 H86 N O8 P	787.608	9.816711	9.7%	9.8%	9.9%	9.3%	9.6%	9.2%	9.3%	9.6%	9.7%	9.6%	10.4%
PC 36:2	C44 H84 N O8 P	785.592	8.810341	4.6%	4.5%	4.6%	4.5%	4.4%	4.4%	4.4%	4.5%	4.4%	4.5%	9.8%
PC 36:4	C44 H80 N O8 P	781.5607	7.57575	1.8%	1.8%	1.8%	1.7%	1.7%	1.7%	1.7%	1.7%	1.7%	1.7%	10.8%
PC 38:1	C46 H90 N O8 P	815.638	11.07277	0.3%	0.2%	0.2%	0.2%	0.2%	0.2%	0.2%	0.2%	0.2%	0.2%	17.6%
PC 38:3	C46 H86 N O8 P	811.6073	9.504333	3.1%	3.0%	3.0%	3.3%	3.2%	3.2%	3.3%	3.3%	3.3%	3.2%	4.5%
PC 38:4	C46 H84 N O8 P	809.5921	8.985689	4.3%	4.2%	4.3%	4.2%	3.6%	3.7%	4.1%	3.7%	4.2%	4.1%	13.3%

PC 38:5	C46 H82 N O8 P	807.5762	7.930222	3.7%	3.8%	3.7%	3.8%	3.7%	3.6%	3.8%	3.8%	3.7%	3.7%	8.4%
PC 38:6	C46 H80 N O8 P	805.5608	7.520356	1.7%	1.8%	1.6%	1.7%	1.7%	1.7%	1.7%	1.6%	1.7%	1.7%	8.7%
PC 38:7	C46 H78 N O8 P	803.5417	7.567773	0.2%	0.2%	0.2%	0.2%	0.2%	0.2%	0.2%	0.2%	0.2%	0.2%	9.9%
PC 40:4	C48 H88 N O8 P	837.6214	10.02341	0.3%	0.3%	0.3%	0.3%	0.3%	0.3%	0.3%	0.3%	0.3%	0.3%	12.6%
PC 40:5	C48 H86 N O8 P	835.6073	9.324326	3.7%	3.6%	3.6%	3.5%	3.5%	3.5%	3.5%	3.5%	3.5%	3.6%	9.9%
PC 40:6	C48 H84 N O8 P	833.5922	8.9232	2.6%	2.6%	2.6%	2.5%	2.5%	2.5%	2.5%	2.6%	2.5%	2.5%	9.8%
PC 40:7	C48 H82 N O8 P	831.5746	7.87575	0.4%	0.4%	0.4%	0.4%	0.3%	0.3%	0.3%	0.4%	0.3%	0.4%	11.5%
PC 40:8	C48 H80 N O8 P	829.5575	7.937091	0.4%	0.4%	0.4%	0.4%	0.4%	0.4%	0.4%	0.4%	0.4%	0.4%	11.1%

PC 40:9	C48 H78 N O8 P	827.5421	7.521023	0.2%	0.2%	0.2%	0.2%	0.2%	0.2%	0.2%	0.2%	0.2%	0.2%	8.9%
PC 42:8	C50 H84 N O8 P	857.5888	9.319705	0.4%	0.4%	0.4%	0.4%	0.4%	0.4%	0.4%	0.4%	0.4%	0.4%	8.5%
PC 42:9	C50 H82 N O8 P	855.5731	8.921886	0.3%	0.3%	0.3%	0.3%	0.3%	0.3%	0.3%	0.3%	0.3%	0.3%	9.0%
Total peak area PC				101464523500.0%	101691028600.0%	103733456000.0%	85848106800.0%	88108395800.0%	88723928200.0%	87282332000.0%	87449832600.0%	87541334100.0%		8.1%
PC O-34:1	C42 H84 N O7 P	745.5954	9.292523	35.5%	34.1%	35.1%	35.2%	35.1%	34.3%	36.0%	34.8%	35.2%	35.1%	8.8%
PC O-34:2	C42 H82 N O7 P	743.5805	9.136795	19.5%	19.9%	20.2%	19.5%	19.4%	19.1%	20.1%	19.0%	19.6%	19.6%	10.2%
PC O-36:5	C44 H80 N O7 P	765.5654	8.251818	6.3%	6.9%	6.1%	6.6%	6.4%	6.5%	7.0%	6.7%	5.8%	6.5%	9.7%
PC O-38:6	C46 H82 N O7 P	791.5806	8.592116	13.6%	14.0%	13.5%	13.5%	13.8%	13.6%	11.6%	13.8%	13.7%	13.5%	11.6%
PC O-38:7	C46 H80	789.5655	8.180186	10.1%	9.6%	9.9%	9.0%	8.9%	9.9%	8.8%	10.0%	10.1%	9.6%	12.8%

	N O7 P													
LPC O-16:0	C24 H52 N O6 P	481.3518	1.344233	4.2%	4.5%	4.3%	4.6%	4.6%	4.6%	4.7%	4.5%	4.5%	4.5%	6.2%
LPC O-16:1	C24 H50 N O6 P	479.3375	1.278152	8.3%	8.9%	8.6%	9.1%	9.4%	9.4%	9.3%	8.8%	8.6%	8.9%	7.1%
LPC O-18:0	C26 H56 N O6 P	509.3831	1.947136	2.4%	2.0%	2.3%	2.4%	2.5%	2.5%	2.6%	2.4%	2.5%	2.4%	7.4%
total peak area PC-O				37077344	36431553	38297478	31188413	31444203	32379792	30439771	31999537	31123191		9.0%
SM 32:1; O2	C37 H75 N2 O6 P	674.5343	4.938023	1.2%	1.1%	1.2%	1.2%	1.2%	1.2%	1.2%	1.2%	1.2%	1.2%	11.1%
SM 33:1; O2	C38 H77 N2 O6 P	688.5499	5.64214	1.6%	1.6%	1.6%	1.6%	1.6%	1.6%	1.6%	1.6%	1.6%	1.6%	11.7%
SM 34:0; O2	C39 H81 N2 O6 P	704.5812	6.918182	2.1%	2.1%	2.1%	2.1%	2.1%	2.1%	2.1%	2.1%	2.1%	2.1%	11.0%
SM 34:1; O2	C39 H79 N2 O6 P	702.5668	6.3374	39.8%	39.6%	39.6%	39.8%	40.0%	39.7%	39.1%	39.5%	39.2%	39.6%	11.1%

SM 34:2; O2	C39 H77 N2 O6 P	700.5503	5.381422	4.2%	4.1%	4.2%	4.2%	4.2%	4.1%	4.2%	4.2%	4.2%	4.2%	10.4%
SM 35:1; O2	C40 H81 N2 O6 P	716.581	7.082163	1.1%	1.1%	1.1%	1.1%	1.1%	1.1%	1.1%	1.1%	1.1%	1.1%	10.5%
SM 36:1; O2	C41 H83 N2 O6 P	730.5974	7.8	7.3%	7.2%	7.4%	7.3%	7.4%	7.5%	7.3%	7.5%	7.3%	7.3%	10.7%
SM 36:2; O2	C41 H81 N2 O6 P	728.5813	6.835907	2.6%	2.5%	2.6%	2.6%	2.6%	2.6%	2.6%	2.6%	2.6%	2.6%	10.1%
SM 38:1; O2	C43 H87 N2 O6 P	758.6271	9.252326	2.3%	2.3%	2.3%	2.3%	2.3%	2.3%	2.3%	2.3%	2.3%	2.3%	11.5%
SM 39:1; O2	C44 H89 N2 O6 P	772.6419	9.938791	0.9%	0.9%	0.9%	0.9%	0.8%	0.8%	0.9%	0.8%	0.9%	0.9%	12.4%
SM 40:1; O2	C45 H91 N2 O6 P	786.6594	10.5664	10.8%	10.8%	10.7%	10.6%	10.7%	10.6%	10.7%	10.6%	10.8%	10.7%	11.4%
SM 41:1; O2	C46 H93 N2 O6 P	800.6741	11.20788	1.9%	2.0%	2.1%	2.0%	2.0%	2.0%	2.1%	2.1%	2.1%	2.0%	10.9%

SM 42:1; O2	C47 H95 N2 O6 P	814.6905	11.80381	6.6%	6.6%	6.6%	6.5%	5.7%	6.2%	6.6%	6.2%	6.2%	6.4%	14.4%
SM 42:2; O2	C47 H93 N2 O6 P	812.6755	10.74195	17.7%	18.0%	17.7%	18.0%	18.2%	18.2%	18.4%	18.2%	18.3%	18.1%	9.7%
total peak area SM				339981497	341220952	352041016	279383063	280651912	289339178	276205763	277143093	273704883		10.9%

Table S10. Data used for the radar plots. The percent relative amount of lipid species within a class, calculated only for lipids detected by all methods (except CE class not detected for SPE method). Only 10 most abundant lipids in FBSEV samples are presented in tables.

Name	LLE	SIN_PH	DI	SPE
CE 14:0	0.7	0.8	0.7	not detected
CE 16:0	1.2	0.6	7.4	not detected
CE 16:1	6.3	7.3	7.8	not detected
CE 18:1	22.4	22.7	25.0	not detected
CE 18:2	22.0	20.4	20.6	not detected
CE 18:3	3.6	3.2	2.8	not detected
CE 20:3	3.9	3.5	3.1	not detected
CE 20:4	25.1	26.7	20.3	not detected
CE 20:5	7.0	7.2	5.1	not detected
CE 22:6	7.6	7.2	6.7	not detected
cholesterol	0.1	0.3	0.4	not detected
LPC 16:0	4.9	7.7	7.9	7.7
LPC 18:0	4.3	6.4	7.2	9.5
LPC 18:1	4.4	6.5	4.9	11.6
PC34:1	19.7	18.2	21.1	15.8
PC 36:1	14.2	11.8	15.0	10.5
PC 36:2	6.1	5.4	3.7	4.9
PC 38:4	6.0	5.2	5.8	4.5
PC 38:5	5.0	5.1	3.8	4.1
PC 40:5	4.9	4.3	4.4	3.9
PC 40:6	4.2	3.7	3.9	2.8
other PCs	26.5	25.7	22.3	24.7
SM 34:1; O2	37.3	41.6	41.2	39.6
SM 34:2; O2	3.8	4.8	3.8	4.2
SM 36:1; O2	7.2	7.2	7.3	7.3
SM 36:2; O2	2.5	2.9	2.5	2.6
SM 40:1; O2	11.4	9.5	11.7	10.7
SM 34:0; O2	2.1	2.2	2.0	2.1
SM 41:1; O2	2.3	1.9	2.2	2.0
SM 42:1; O2	7.0	5.4	7.0	6.4
SM 42:2; O2	19.3	17.0	15.4	18.1
SM 38:1; O2	2.3	2.2	1.8	2.3
other SMs	4.9	9.7	5.0	4.7
